# Helcococcus ovis in a patient with an artificial eye: a case report and literature review

**DOI:** 10.1186/s12879-018-3310-7

**Published:** 2018-08-14

**Authors:** Liyan Mao, Zhongju Chen, Yanfang Lu, Jing Yu, Yu Zhou, Qun Lin, Ying Luo, Ziyong Sun

**Affiliations:** Department of Laboratory Medicine, Tongji Hospital, Tongji Medical College, Huazhong University of Science and Technology, Wuhan, 430030 China

**Keywords:** Eye infection, Artificial eye, *Helcococcus ovis*, 16S rRNA gene sequencing, Antimicrobial susceptibility

## Abstract

**Background:**

*Helcococcus ovis*, belonging to the genus of *Helcococcu* in *Peptostreptococcaceae,* is one kind of facultative anaerobic and gram-positive cocci, which was first isolated from a mixed infection in sheep in 1999. To our knowledge, it’s known as an invasive pathogen in animals, and never been reported as a human pathogen in published literature. The aims of this work are to describe the first report of *H. ovis* which was recovered from the artificial eye of human case and perform a literature review*.*

**Case presentation:**

A 26 year-old man reporting pyogenic infection with an artificial eye attended ophthalmic ward in Tongji hospital. After physical examination, clinical and laboratory investigations, the diagnosis of eye infection caused by *Helcococcus ovis* and *Staphylococcus aureus* was established. Receiving a medico-surgical approach, the patient was successfully treated. The treatment consisted in intravenous cefotaxime and ornidazole, levofloxacin eye drops during two weeks and removing of right artificial eye with debridement.

**Conclusions:**

We describe here the first known case of *H. ovis* which was recovered from human artificial eye. This report different from previous data found in the literature emphasizes the invasive potential of this bacterial species as a pathogen in human. Prospectively, the application of next generation sequencing tools would contribute to a more accurate classification of clinical strains.

**Electronic supplementary material:**

The online version of this article (10.1186/s12879-018-3310-7) contains supplementary material, which is available to authorized users.

## Background

*Helcococcus,* a facultatively anaerobic, catalase-negative, Gram-positive cocci, was first described in 1993 by Collins and colleagues [1]. This genus composes of five species, namely *Helcococcus kunzii* (first reported in 1993 [1])*, Helcococcus ovis* (first reported in 1999 [2])*, Helcococcus pyogenica* (first reported in 2004 [3]), *Helcococcus sueciensis* (first reported in 2004 [4]) and *Helcococcus seattlensis* (first reported in 2014 [5])*.* All species, with the exception of *H. ovis,* have been isolated from human specimens [3–8], and *H. kunzii* is the most common pathogen [7–10]. *H. ovis* was first isolated from a mixed infection in sheep in 1999 and was subsequently reported in bovine, horses and goats [2], but has never been isolated from human specimens, even as a result of foreign body invasion [11, 12]. In this report, we describe the first known human case of artificial eye infection, which *H. ovis* was isolated from the artificial eye*.*

## Case presentation

A 26 year-old man attended our ophthalmic ward in April 2017 with intermittent bleeding of the right eye, from which there was also strong odor. The patient was a heavy smoker but had no other underlying conditions. He had no history of drug-use. From his medical history it was noted that the patient had undergone a right ophthalmectomy 24 years previously due to retinoblastoma, and implantation of an artificial right eyeball in 2014 (timeline shown in Additional file 1).

On admission, his pulse rate was between 80 and 100 beats/min. His body temperature and respiratory rate were both normal. Physical examination showed narrow conjunctival sac in right eye and the exposure of ocular prosthesis, which was discharging a yellow-green secretion along with a strong odor. The visual acuity of left eye was 0.3, and the intraocular pressure was 15 mmHg. All other characteristics of the left eye were normal. A auscultation did not show any abnormality in the lungs, and no signs of carotid murmur were found. Interestingly, laboratory investigations did not reveal abnormal inflammatory markers such as leukocytosis or any increase in neutrophils or C-reaction protein. According to clinical and laboratory investigations, infectious endocarditis was not suspected. The patient had no history of other immunosuppressive conditions, except smoking and a retinoblastoma 24 years previously. The patient did not report any direct contact with animals; however, he did work in a clothing factory so would have been contact with wool and cowhide for one month of the year. Three months had elapsed between the patient last coming into contact with wool and cowhide and the appearance of clinical symptoms. Considering the results of these investigations, partial artificial eye infection, especially anaerobic organism infection, was suspected.

**Fig. 1:**
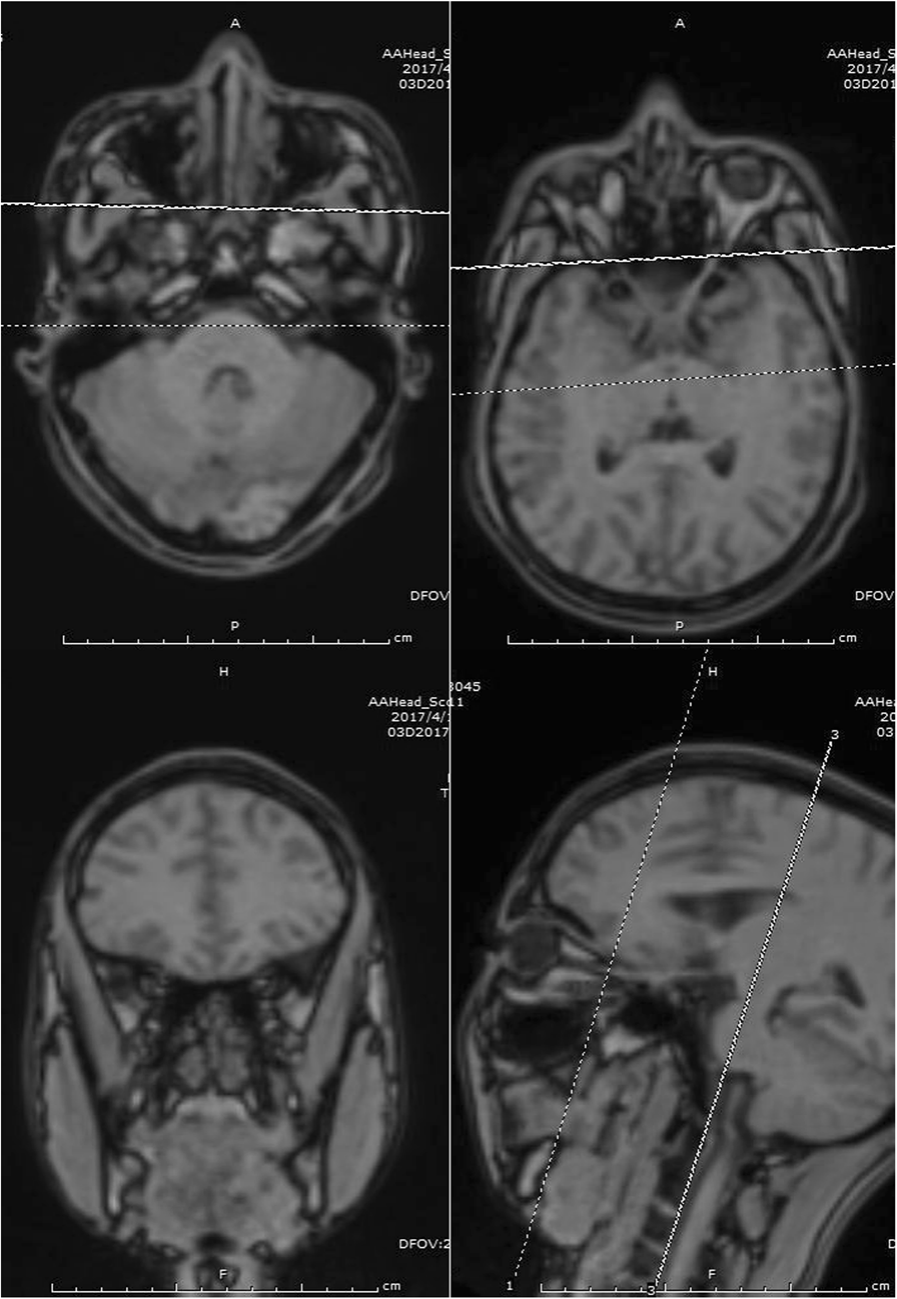
Eye magnetic resonance imaging of the patient

Imaging workups were completed, which included chest x-ray, transthoracic echocardiography and eye magnetic resonance imaging. As shown in Fig. 1, eye magnetic resonance imaging revealed that the tissue surrounding the right eye prosthesis as well as the soft tissue of the lacrimal gland area were swollen, whereas the left eye appeared normal. Inflammatory disease in the right eye was therefore suspected. According to chest x-ray and transthoracic echocardiography, no obvious abnormalities in the lungs or heart were observed.

**Fig. 2:**
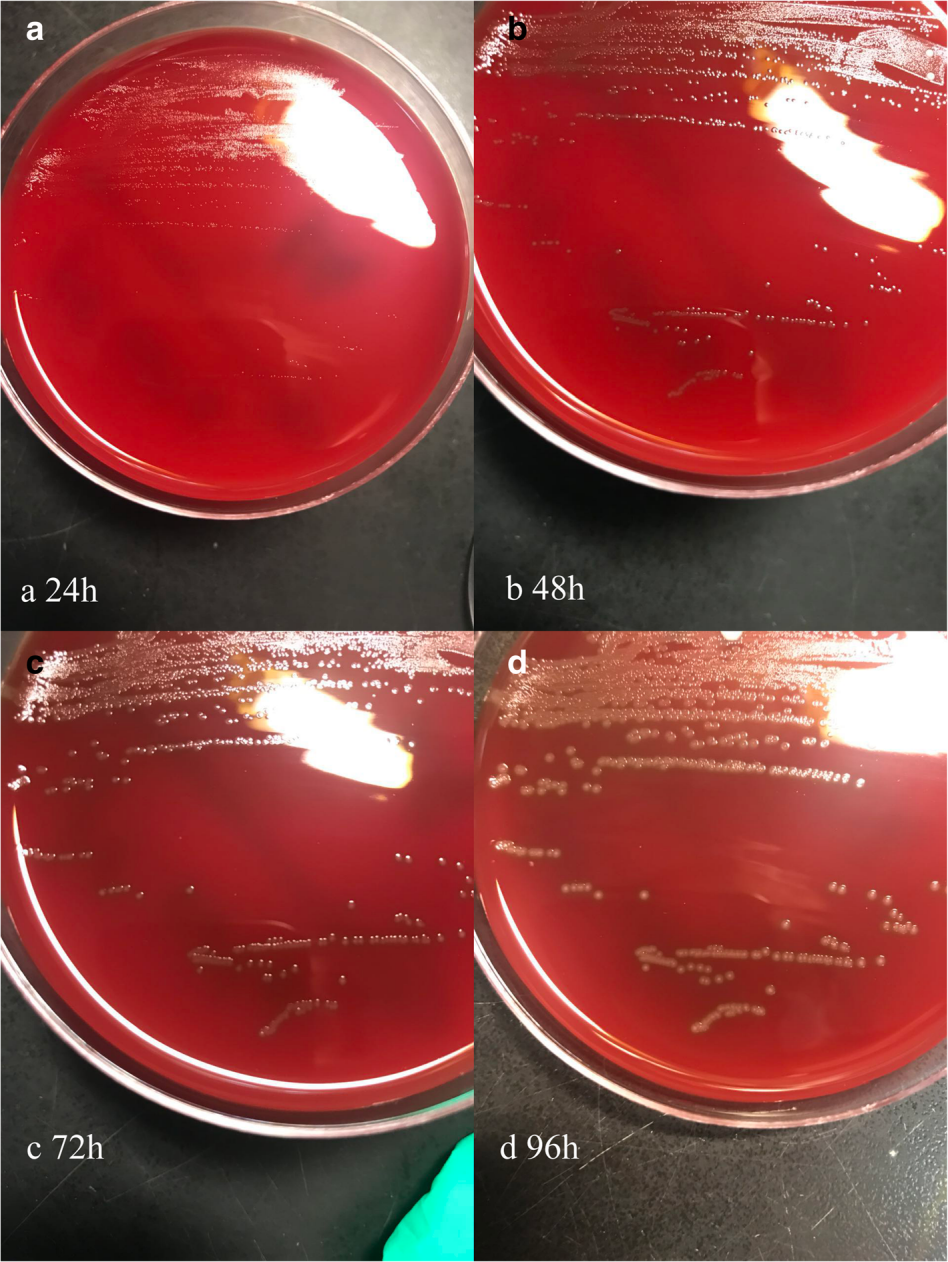
Colonies of pure *Helcococcus ovis* on Columbia agar plates supplemented with 5% sheep blood. **a** 24 h after incubation; (**b**) 48 h after incubation; (**c**). 72 h after incubation; (**d**) 96 h after incubation

**Fig. 3:**
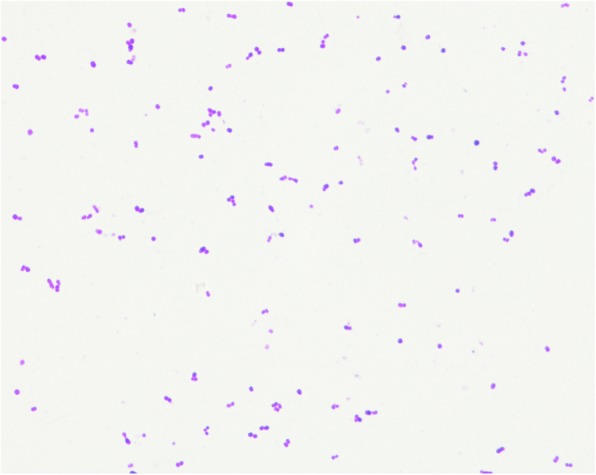
Gram staining of *Helcococcus ovis* under oil microscope

Before surgery, a few specimens of the right eye secretions were collected to be cultured, but no bacteria were isolated, possibly because most of the secretions had been absorbed by the artifical eye making it yellow-green in appearance. After removal of the right artificial eye with debridement (5 days after admission), both the artificial eye and specimens of the eye secretions were sent for bacterial culture under aerobic and anaerobic conditions. No bacterial growth was detected from the ophthalmic secretions, but cultures were obtained from the artificial eye. Sparse growth of β-hemolytic cocci and heavy growth of small, non-hemolytic, translucent colonies were observed on Columbia agar plates supplemented with 5% sheep blood (BioMérieux, Marcy l’Etoile, France) under aerobic conditions after 48 h. And the latter colonies only grow close to the hemolysis zone of the former one. Under anaerobic conditions, only the small, translucent colonies were detected from the artificial eye (as shown in Fig. 2). Of the two colony types, the β-hemolytic cocci were confirmed as *Staphylococcus aureus,* whereas the small, translucent colonies stained positive in a Gram stain and occurred singly, in pairs, or in short chains (Fig. 3). Catalase and oxidase reactions of the unknown colonies were negative and phenotypic characterization using the Vitek2 GP system (BioMérieux) was inconclusive. However, Matrix-assisted laser desorption/ionization time of flight (MALDI-TOF) mass spectrometry revealed a match with *Helcococcus ovis* DSM 21504 T DSM (log score: 1.637) according to the Brucker Maldi-Biotyper database. Identification of this organism was confirmed by 16S rRNA gene sequencing. BLAST analysis of the partial 16S rRNA gene sequence derived from our isolate (1492 nucleotides, deposited in the GeneBank database under accession number MG188744) showed 98.9% identity (15 nucleotide differences) with the 16S rRNA gene sequence of H. ovis s840–96-2 deposited in the GenBank database under accession number NR027228 by Collins and coworkers [2] in 1999 when this species was first described.

Antimicrobial susceptibility testing (AST) of both strains was performed. The disk diffusion method was carried out and with the exception of penicillin, erythromycin and clindamycin, *S. aureus* isolated from this case was susceptible to all other drugs including cephalosporins and fluoroquinolones. AST for *Helcococcus* was performed using the CLSI broth microdilution method on Mueller-Hinton II broth (BD Diagnostics, Heidelberg, Germany) supplemented with 3% (vol/vol) lysed horse blood (Oxoid, Wesel, Germany) and 0.001% (wt/vol) pyridoxal HCl (Sigma–Aldrich, Munich, Germany) incubated at 37 °C in 5% CO2 for 24 h [13, 14]. *Streptococcus pneumoniae* ATCC 49619 served as a quality control. Then, we changed the method and performed an E-test on blood agar, with *S. aureus ATCC 29213* as the quality control (for reference only). The MICs (μg/L) of the drugs for this strain are reported in Table 1. Since no antimicrobial testing guidelines are currently available from the Clinical and Laboratory Standards Institute (CLSI) for *Helcococcus*, the MICs were determined in reference to the CLSI guidelines for *S. aureus* [5]. According to the CLSI [5, 15], this strain was susceptible to penicillin, ampicillin, teicoplanin, ceftriaxone, vancomycin, and linezolid.

**Table 1:** Antimicrobial susceptibilities of *Helcococcus ovis* in different studies

Author	method	Penicillin G	Amoxicillin	Amoxicillin + clavulanic acid	Ampicillin	cephalothin	ceftazidime	Clindamycin	Vancomycin	Metronidazole	Erythromycin	Tetracycline
Collins,1999 [2]	N.A	/	/	/	/	/	/	/	S	/	/	/
Post, 2003 [18]	N.A	/	/	/	/	/	/	/	S	/	/	/
Rothschild, 2004 [16]	Etest strips	S (< 0.016)	/	/	/	/	/	S(0.125)	/	R(> 256)	/	/
Kutzer, 2008 [17]	N.A	/	/	/	/	/	/	/	S	/	/	/
Bilk, 2011 [13]	Broth microdilution, resistance genes	S (< 0.25)	S (< 0.5)	S (< 0.5)	/	S (< 0.5)	/	S(< 0.12)	/	/	10% R(> 8)	83% R(> 8)
García,2012 [20]	modified K–B	/	S	/	S	S	S	/	S	/	/	I
Our study [21]^a^	Etest strips	S(0.064)	/	/	S(0.016)	/	/	/	S(0.19)	/	/	/

Note: *NA* means Not Available, the method of AST was not mentioned. / means this drug was not tested

^a^In our study, we also tested some other drugs: TEC(0.047,S); LZD(1.5,S); CXO(0.094,S)

After admission, the patient received levofloxacin eye drops 4 times per day until being discharged from hospital. After surgery, the patient was initially treated with intra-venous cefotaxime (2.25 g/250 ml NaCl, 1/day) and ornidazole (500 mg/day) for 1 week. Two weeks after admission, the patient recovered and was discharged from hospital. Six months later, the patient returned to the hospital to finish implantation of the artificial right eyeball and no signs of infection were detected. With the patient’s consent, we collected samples from the skin around both eyes for aerobic and anaerobic culture, but only normal skin flora were detected, such as coagulase-negative Staphylococcus. After surgery, the patient was in good health and was discharged from the hospital.

## Discussion and conclusions

*H. ovis* was first described by Collins and colleagues in 1999. Two strains, *He.ovis* CCUG 374411 and CCUG 39041, were isolated as part of a mixed infection from sheep. The first was from the lungs, liver and spleen at necropsy, whereas the second was from the milk of sheep with subclinical mastitis [2]. Compared with the type strain, *Helcococcus kunzii,* which was the first member of the *Helcococcus* genus discovered in 1993 [1], these isolates from sheep were different both in phenotype and genotype, and their sequences were approximately 4% divergent from that of *H. kunzii*. Therefore, it was suggested that the isolates from sheep be classified as a new species, *H. ovis*.

Briefly, *H. ovis* is a facultatively anaerobic, catalase-negative Gram-positive cocci, that is non-motile and whose cells occur singly, in pairs, or in short chains. The organism only grows close to the hemolysis zone of S. aureus’s colonies on blood agar. After sub-culturing, it produces pinpoint, non-hemolytic, non-pigmented colonies without *S. aureus*. In our study, after 48 h incubation (other publication claimed after 72 h incubation on blood agar [16]), this organism showed slight alpha-hemolytic activitiy (as shown in Fig. 2b). There was no difference in growth under 5% CO_2_ or anaerobic conditions.

**Fig. 4:**
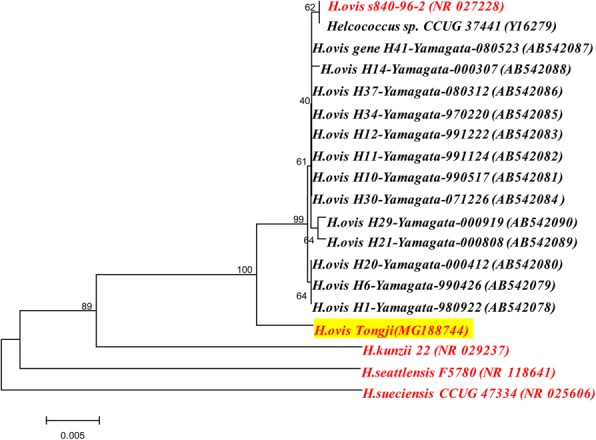
Phylogenetic tree of *Helcococcus ovis* and other Helcococcus genus. Other compared Helcococcus genus include *Helcococcus kunzii*, *Helcococcus sueciensis* and *Helcococcus seattlensis*, and only type strains are focused. The length of 16S rRNA gene sequence of *Helcococcus pyogenica* on the GenBank is only nearly 500 bp, which is not used for comparison. The lengths of all sequences included in the Phylogenetic tree are almost 1400 bp

Biochemical methods are not reliable for *H. ovis* identification. Several studies have reported the misidentification of *H. ovis* as *Granulicatella adiacens* by various biochemical methods [12, 16, 17]*.* In this particular case, phenotypic characterization using the Vitek2 GP system gave an inconclusive result. However, the biochemical results with *H. ovis* in our study showed a marked disparity with the results of *H. ovis* CCUG 374411 and CCUG 39041 (as shown in Table 2, in which only different results are shown). To confirm this disparity, further experiments are needed. MALDI-TOF mass spectrometry was first used to identify *H. ovis* at the species level in our study*.* It presented a low log score value of 1.637, but the matching strain result (*H. ovis* DSM 21504 T DSM) provided a valuable reference. This was due to the limited data in the MALDI-TOF database (Maldi-Biotyper database), which does not contain data on the various subspecies of *H. ovis*. In the future, the MALDI-TOF databases are expected to be expanded. 16S rRNA gene sequencing confirmed the species level identification in our study and we deposited the sequence in the GenBank database under accession number MG188744. Compared with the original sequence deposited by Collins and colleagues under Genbank accession number NR027228, our sequence differed by 15 base pairs. We also mapped phylogenetic tree based on data obtained from the GenBank database (1400 bp of sequence) using the maximum likelihood method (MEGA, version 6.0)(all original sequence data see Additional file 2). As shown in Fig. 4, *H. ovis Tongji* identified in our study was closely related to *H. ovis*, but differed from other *H. ovis* strains. According to the phenotype, genotype and clinical significance of our isolate, we consider it might to be a new subspecies of *H. ovis*. With the rapid development of whole genome sequencing, new genomic tools will help to minimize error and ensure accurate identification of bacterial species.

**Table 2:** Biochemical results for *H. ovis* isolate compared to results obtained for *H. ovis* control strains

Phenotypic characteristic	*H. ovis* control strains^a^		H. ovis in our study
	CCUG 37441	CCUG 39041	
β-Galactosidase	+	+	–
α-Glucosidase	+	+	–
Cyclodextrin	–	+	+
Leucine aminopeptidase	+	+	–
β-Glucuronidase	+	+	–
sorbitol	–	–	+
N-Acetyl-D-Glucosaminidase	–	–	+
Mannitol	–	–	+
Methyl-β-D-glucopyranoside	–	–	+
Pullulan	–	–	+
Raffinose	–	–	+
Trehalose	–	–	+
Arginine dihydrolase	–	–	+

Note: ^a^We didn’t test *H. ovis* control strains, and the results of *H. ovis* control strains came from the results of Collins’ and Kutzer’s studies

This article also reviewed previous literature about *H. ovis* infection published by several authors. The main clinical and microbiological characteristics of *H. ovis* infections are shown in Table 3. In 1999, this bacterium was first recovered from a mixed infection in sheep, but at this time the clinical significance of *H. ovis* was then unknown [2]. In 2003, Post and colleagues first described the isolation of *H. ovis* from cattle. Most anaerobes were assumed to grow polymicrobially. However, when in combination with *Escherichia coli*, *H. ovis* was associated with lesions in multiple tissues suggesting an etiological role in valvular endocarditis [18]. In 2004, relatively pure culture of *H. ovis* from both abscess and transtracheal wash samples indicated that this organism may pathogenic in the lungs of horses [16]. In 2008, 55 cases of bovine valvular endocarditis were collected and *H. ovis* (18, 33%) represented the second most common isolate recovered mainly as a pure culture [17]. This high level of prevalence demonstrated that *H. ovis* was an emerging pathogen in bovine valvular endocarditis. In 2009, *H. ovis* was isolated from a sheep [19], where is was proposed to be the primary pathogen of pleuritis and bronchopneumonia. In 2012, García and coworkers reported that *H. ovis* was the dominant organism isolated from the lungs of a goat with pulmonary abscesses and purulent bronchopneumonia, again suggesting that *H. ovis* may play an etiologic role [20]. In the same year, Schwaiger proposed that the detection of *H. ovis* in four samples might indicate the involvement of this species in the pathogenesis of bovine mastitis [11]. In 2013, both *H. ovis* and *H. kunzii* were isolated from daily cows where they were potentially involved in uterine infections [12]. In 2014, *H. ovis* was isolated as a pure culture from the stomach contents of an aborted fetus [21]. This case was thought to be the first indication that *H. ovis* may cause bovine abortion. As shown in Table 3, all isolates of *H. ovis* recovered to date were from animals i.e. sheep, bovine, horses and goats, where they caused infections such as valvular endocarditis, pulmonary abscess, pleuritis and bronchopneumonia, mastitis, and abortion. Furthermore, all isolates of *H. ovis* published to date had similar characteristics with virtually all being sensitive to vancomycin and sharing sequences similar (no more than 5 bp differences) to that of the type strain, *H. ovis* CCUG 37441.

**Table 3:** Main features of reported cases of *Helcococcus ovis* infections

Author	Infected animal	Helcococcus ovis	Diagnosis	Isolation source	Treatment	Outcome	Other Bacteria	Methods of identification
Collins,1999^a^ [2]	sheep^1^; sheep^2^	CCUG 37441(s840–96-2)^1^; CCUG 39041^2^	N.A.^1^;subclinical mastitis^2^	lung, liver and spleen.^1^; milk of sheep^2^	N.A.	1: death; 2:N.A.	*Arcanobacterium pyogenes*^1^; *Staphylococcus spp*^2^.	-API rapid ‘ID32 strep and API ZYM systems (bioMerieux) -PFGE -16 s rRNA gene sequencing
Post,2003 [18]	bovine	one single base change vs CCUG 37441	valvular endocarditis	atrioventricular heart valve, lung, liver	Various antimicrobial agents (details N.A.)	death	*Escherichia coli*	-16S rRNA gene sequencing
Rothschild, 2004 [16]^b^	horse	one single base change (99.93%) vs CCUG 37441	pulmonary abscess	abscess fluid^1^; transtracheal wash^2^	penicillin, gentamicin, enrofloxacin(whole 8 months); trimethoprim-sulfadiazine (later 2 months)	1: recover but recurring pleuropneumonia; 2: recovering well.	none^1^; *Pseudomonas spp*^2^	-API 20 Strep kit (Biomerieux)-16S rDNA gene sequencing
Kutzer, 2008 [17]	Bovines(55)^c^	at least 99.7% vs CCUG 37441	valvular endocarditis	hearts	N.A.	1 death; other N.A.	16 none; 1 *Pseudomonas aeruginosa*,*Enterococcus faecalis*; 1 *Streptococcus dysgalactiae*.	-16S rRNA gene sequencing -API rapid ID 32 Strep and API ZYM kits (bioMe’rieux, Nu¨rtingen, Germany) -Fluorescence in situ hybridization (FISH)
Zhang, 2009 [19]	sheep	100% match vs CCUG 37441	pleuritis and bronchopneumonia	lung tissue	N.A.	death	*Nocardia spp*.; *Bacillus spp*; *Staphylococcus aureus*	-16S rDNA gene sequencing
Bilk, 2011 [13]	bovine(29)^d^	at least 99.4% vs CCUG 37441	18 valvular endocarditis; 7 metritis and/or bortions; 3 bronchopneumonia; 1 ulcerative glossitis	N.A.	N.A.	N.A.	N.A.	-16S rRNA gene sequencing -API rapid ID 32 Strep and API ZYM kits (bioMe’rieux, Nu¨rtingen, Germany) -Fluorescence in situ hybridization (FISH)
García,2012 [20]	goat	H41-Yamagata-080523 (one single base change vs CCUG 37441)	purulent bronchopneumoniaand pulmonary abscesses	lung tissues	tetracycline	death	none	-16S rRNA gene sequencing
Schwaiger,2012 [11]	bovine	N.A.	mastitis	milk	N.A.	N.A.	*A. pyogenes*, *Peptoniphilus/Peptostreptococcus*	-VITEK system (bioMérieux Deutschland GmbH, Nürtingen, Germany). -PCR-single strand conformation polymorphism
Locatelli,2013 [12]	bovine	1105 (99%,single base change CCUG 37441)	puerperal metritis	N.A.	N.A.	N.A.	*Escherichia coli*	-API Systems (bioMérieux, Marcy l..Etoile, France). -16S rRNA gene sequencing
AHVLA^e^, 2014 [21]	bovine	N.A.	abortion	stomach contents	N.A.	fetus death	none	-16S rRNA gene sequencing

Note: *NA* means Not Available, not mentioned in original article

^a^Collins’s study contained two sheeps; 1 means the first sheep, while 2 means the other one. ^b^Rothschild’s study mentioned one horse, but two isolates of *Helcococcus ovis* were recovered from this horse. 1 means the first time that *H.ovis* was isolated from abscess fluid, while 2 means *H.ovis* was isolated from transtracheal wash. ^c^55 cases of bovine were collected in Kutzer’s study. And 18 strains of *Helcococcus ovis* were isolated. ^d^29 means the number of the bovine in Bilk’s study, and 29 strains of *Helcococcus ovis* were isolated. ^e^Animal Health and Veterinary Laboratories Agency’s (AHVLA’s) disease surveillance report

To the best of our knowledge, we report here the first known human infection of *H. ovis* in a patient with an artificial eye. Although *H. ovis* was recovered along with *S. aureus,* dominant growth of *H. ovis* was detected in the artificial eye*.* In addition*,* considering the yellow-green appearance and the strong odor of the ocular prosthesis, anaerobic infection was more suspected. Therefore, we concluded that *H. ovis* might be the primary pathogen responsible for the eye infection. Interestingly, the patient reported no direct contact with animals, however, his work in a clothing factory brought him into contact with wool and cowhide for one month of the year. Three months had elapsed between the patient’s last contact with wool and cowhide and the development of symptoms. Furthermore, neither his work colleagues nor relatives reported any similar infections. Although skin samples from the patient were also cultured, no significant growth was detected. The origin of this infection remains unclear and was not assessed by any microbiological data (i.e., we did not assess the patient’s working and living environments). The patient did not report any recent drug use before admission, and according to physical examination and laboratory investigations, he was in relatively good health condition. Although he was a smoker and had a history of ocular surgery. Compared with the isolates of *H. ovis* previously reported in the literature, our isolate displayed distinct clinical and biochemical characteristics as well as genotypic differences (15 bp differences from the type strain). According to these data and the phylogenetic tree, we propose that our isolate might represents a new subspecies of *H. ovis*; however, further studies will be required to confirm this hypothesis.

We describe here the first known case of *H. ovis* in a young adult with an artificial eye infection. This case emphasizes the previously unreported invasive potential of this bacterial pathogen. Prospectively, the application of next generation sequencing tools would contribute to a more accurate classification of clinical strains.

## Additional files

Additional file 1: Timeline. The timeline has covered this patient’s relevant medical history, and the whole procedures during the period of the hospital. (DOC 42 kb)

Additional file 2: Sequence data. This file is divided into three parts. Part one is the 16S rRNA gene sequence of *H. ovis* in this article. Part two contains the sequences of type strains of *Helcococcus kunzii*, *Helcococcus sueciensis* and *Helcococcus seattlensis.* Part three contains all other sequences of *H. ovis* that have been published in other articles. (DOC 80 kb)

## Abbreviations

AST: Antimicrobial susceptibility testing;
CLSI: Clinical and Laboratory Standards Institute
MALDI-TOF: Matrix-assisted laser desorption/ionization time of flight
Spn: *Streptococcus pneumoniae*
