# Supplementary material for: Helcococcus ovis in a patient with an artificial eye: a case report and literature review
Source: BMC Infect Dis. 2018 Aug 14;18:401. doi: 10.1186/s12879-018-3310-7 (PMC6092862; doi:10.1186/s12879-018-3310-7)
Supplement: Supplementary file 1 — Timeline. The timeline has covered this patient’s relevant medical history, and the whole procedures during the period of the hospital. (DOC 42 kb) [file 12879_2018_3310_MOESM1_ESM.doc]

**1993：Right ophthalmectomy (retinoblastoma)**

**2014：Right artificial eyeball implantation**

**2016-11:** **Wools and cowhides contact history**

**2017-4-12**

**Current Illness:** right eye intermittent bleeding with cacosmia

**Physical Examination**

**Recover**

**2017-4-17**

**Surgery: removing of right artificial eye with debridement**

**Medicine: intravenous cefotaxime and ornidazole**

**Diagnostic Evaluations:** suspected anaerobic infection in right eye

**Levofloxacin eye drops**

**2017-4-24**

**Diagnose:** infection primarily caused by *Helcococcus ovis*

**Discharge from hospital**

**2017-10-11**

**Surgery: right artificial eyeball implantation**
