# Supplementary material for: Helcococcus ovis in a patient with an artificial eye: a case report and literature review
Source: BMC Infect Dis. 2018 Aug 14;18:401. doi: 10.1186/s12879-018-3310-7 (PMC6092862; doi:10.1186/s12879-018-3310-7)
Supplement: Supplementary file 2 — Sequence data. This file is divided into three parts. Part one is the 16S rRNA gene sequence of H. ovis in this article. Part two contains the sequences of type strains of Helcococcus kunzii, Helcococcus sueciensis and Helcococcus seattlensis. Part three contains all other sequences of H. ovis that have been published in other articles. (DOC 80 kb) [file 12879_2018_3310_MOESM2_ESM.doc]

Part One: *H. ovis* in this article

>gi|1254399306|gb|MG188744.1| Helcococcus ovis strain Tongji 16S ribosomal RNA gene, partial sequence

GAGTTTGATCCTGGCTCAGGACGAACGCTGGCGGCGTGCTTAACACATGCAAGTTGAACGAGAATTTTTT

AATAGATTTCTTCGGAATGAAATTAAAAGAGGAAAGTAGCGAACGGGTGAGTAACACGTGAGAAACCTGCCTTTCACAAAGGGATAGCCTCGGGAAACCGGGATTAATACCTTATGACACGGAGATATCGCATGATAATTTCGTTAAAGAATTTCGGTGAAAGATGGTCTCGCGTCTGATTAGCTAGTTGGTAAGGTAACGGCTTACCAAGGCGACGATCAGTAGCCGGATTGAGAGGTTGAACGGCCACACTGGAACTGAGACACGGTCCAGACTCCTACGGGAGGCAGCAGTGGGGAATTTTGCACAATGGGGGGAACCCTGATGCAGCGACGCCGCGTGAACGATGAAGGTCTTCGGATTGTAAAGTTCTGTCCTTAGTGAAGATAATGACTGTAACTAAGAAGCAAGCCCTGGCTAAATACGTGCCAGCAGCCGCGGTAATACGTATGGGGCAAGCGTTGTCCGGAATTATTGGGCGTAAAGGGTACGTAGGCGGTAATTTAAGTCTGAATTAAAAGGCTGTGGCTCAACCATAGTAAGGTTCAGATACTGGATTACTTGAGTAGATGAGGGGAAAGTGGAATTCCATGTGTAGCGGTGAAATGCGTAGATATATGGAGGAACACCTGTGGCGAAGGCGACTTTCTGGAATCTAACTGACGCTGAGGTACGAAGGCGTGGGGAGCAAACAGGATTAGATACCCTGGTAGTCCACGCAGTAAACGATGAGTGCTAGTTGTCGGGAGTCAAATCTCGGTGACGCAGCTAACGCATTAAGCACTCCGCCTGGGGAGTACGTACGCAAGTATGAAACTCAAAGGAATTGACGGGGACCCGCACAAGCAGCGGAGCATGTGGTTTAATTCGAAGCAACGCGAAGAACCTTACCAAGGCTTGACATATACAGGAATATGCTAGAGATAGTATAGTTTCTTCGGAAACTTGTATACAGGTGGTGCATGGTTGTCGTCAGCTCGTGTCGTGAGATGTTGGGTTAAGTCCCGTAACGAGCGCAACCCTTATCTTTAGTTACCAGCATTTCGGATGGGGACTCTAGAGAGACTGCCGGTGATAAACCGGAGGAAGGTGGGGATGACGTCAAATCATCATGCCCTATATGTCTTGGGCTACACACGTGCTACAATGGTCTGAACAAAGCGTAGCTACCTCGTGAGAGCAAGCGAATCGCATAAAACAGATCTCAGTTCGGATTGTAGGCTGCAACTCGCCTACATGAAGTCGGAGTTGCTAGTAATCGTGGATCAGAACGCCACGGTGAATGCGTTCCCGGGTCTTGTACACACCGCCCGTCACACCATGGGAGTTGGCAATACCCGAAGCCGTCGAGCTAACCGTTAGGAGGCAGACGTCGAAGGTAGGGTCAATAACTGGGGTGAAGTCGTAACAAGGTAGCCGTAA

Part Two: type strains

>gi|645321746|ref|NR_118641.1| Helcococcus seattlensis strain F5780 16S ribosomal RNA gene, partial sequence

TTTTGGAGAGTTTGATCCTGGCTCAGGACGAACGCTGGCGGCGTGCTTAACACATGCAAGTTGAACGAGA

ATTTTTYTAGAGAWTTCTTCGGAATGATCCATAGAAAAAGGAAAGTAGCGAACGGGTGAGTAACACGTGAGAAACCTGCCTTTCACAAAGGGATAGCTTCGGGAAACCGGAATTAATACCTTATGACACATAARTATCGC

ATGATAAATATGTTAAAGAATTTCGGTGAAAGATGGTCTCGCGTCTGATTAGCTAGATGGTGGGGTAATG

GCCTACCATGGCGATGATCAGTAGCCGGATTGAGAGGTTGAACGGCCACACTGGAACTGAGACACGGTCCAGACTCCTACGGGAGGCAGCAGTGGGGAATTTTGCACAATGGGGGAAACCCTGATGCAGCGACGCCGCGTGAATGATAGAAGGCCTTCGGGTTGTAAAATTCTGTCCTTAGTGAAGATAATGACAGTAACTAAGAAGCAAGCCCTGGCTAAATACGTGCCAGCAGCCGCGGTAATACGTATGGGGCAAGCGTTGTCCGGAATTATTGGGCGTAAAGGGTACGTAGGCGGTTATTTAAGTCCTAATTAAAAGGCAATGGCTCAACCATTGTAAGGTTAGGAAACTAGATAACTTGAGTAGATGAGGGGAAAGTGGAATTCCATGTGTAGCGGTGAAATGCGTAGATATATGGAGGAACACCTGTGGCGAAGGCGACTTTCTGGAATCAAACTGACGCTGAGGTACGAAGGCGTGGGGAGCAAACAGGATTAGATACCCTGGTAGTCCACGCAGTAAACGATGAGTGCTAGTTGTCGGGAGTCAAATCTCGGTGACGCAGCTAACGCATTAAGCACTCCGCCTGGGGAGTACGTGCGCAAGCATGAAACTCAAAGGAATTGACGGGGACCCGCACAAGCAGCGGAGCATGTGGTTTAATTCGAAGCAACGCGAAGAACCTTACCAAGGCTTGACATATACATGGCCGGTGTAGAGATACACCTTTTCTTCGGAACATGTATACAGGTGGTGCATGGTTGTCGTCAGCTCGTGTCGTGAGATGTTGGGTTAAGTCCCGTAACGAGCGCAACCCCTATCTTTAGTTGCTAGCAGGTAAAGCTGAGAACTCTAGAGAGACTGCCGGTGATAAACCGGAGGAAGGTGGGGATGACGTCAAATCATCATGCCCTATATGTCTTGGGCTACACACGTGCTACAATGGTCTGAACAAAGTGCAGCGAACTCGTGAGAGCAAGCGAATCACATAAAACAGATCTCAGTTCGGATTGTAGGCTGCAACTCGCCTACATGAAGTCGGAGTTGCTAGTAATCGTGGATCAGAATGCCACGGTGAATGCGTTCCCGGGTCTTGTACACACCGCCCGTCACACCATGGAAGTTGGCAATACCCGAAGCCGTCGAGCTAACCAATAGGAGGCAGACGTCGAAGGTAGGGTCAATAACTGGGGTGAAGTCGTAACAAGGTAGCCGTATCGGAAGGTGCGGCTGGATCACCTCCTTA

>gi|219878467|ref|NR_025606.1| Helcococcus sueciensis strain CCUG 47334 16S ribosomal RNA gene, partial sequence

TTTGATCCTGGCTCAGGACGAACGCTGGCGGCGTGCTTAACACATGCAAGTTGAACGTGATTTTATAGAC

AGATTTCTTCGGAATGACGATTATAAATGAAAGTAGCGAACGGGTGAGTAACACGTGAGAAACCTGCCTT

TCACAAAGGGATAGCCTCGGGAAACCGGGATTAATACCTTATGATACATAGAAATCGCATGATANCTATG

TTAAAGATTTTATCGGTGAAAGATGGTCTCGCGTCTGATTAGCTAGTTGGTGGGGTAAAGGCCTACCAAG

GCGATGATCAGTAGCCGGATTGAGAGGTTGAACGGCCACACTGGAACTGAGACACGGTCCAGACTCCTACGGGAGGCAGCAGTGGGGAATTTTGCACAATGGGGGAAACCCTGATGCAGCGACGCCGCGTGTACGATGAAGATCTTCGGATTGTAAAGTACTGTCCTTGGTGAAGATAATGACTGTAACCAAGAAGCAAGCCCTGGCTAAATACGTGCCAGCAGCCGCGGTAATACGTATGGGGCAAGCGTTGTCCGGAATTATTGGGCGTAAAGGGTACGTAGGCGGTTATTTAAGTCTAAATTAAAAGGCATTGGCTCAACCAATGTAAGGTTTAGATACTGGATAACTTGAGTAGATGAGGGGAAAGTGGAATTCCATGTGTAGCGGTGAAATGCGTAGATATATGGAGGAACACCAATGGCGAAGGCAACTTTCTGGAATCAAACTGACGCTGAGGTACGAAGGCGTGGGGAGCAAACAGGATTAGATACCCTGGTAGTCCACGCAGTAAACGATGAGTGCTAGATGTTGGGAGTCAAATCTCAGTGTCGCAGCTAACGCAATAAGCACTCCGCCTGGGGAGTACGTACGCAAGTATGAAACTCAAAGGAATTGACGGGGACCCGCACAAGCAGCGGAGCATGTGGTTTAATTCGAAGCAACGCGAAGAACCTTACCAAGGCTTGACATATACATGAAAATCCTAGAGATAGGATCCTCTCTTCGGAGACGTGTATACAGGTGGTGCATGGTTGTCGTCAGCTCGTGTCGTGAGATGTTGGGTTAAGTCCCGTAACGAGCGCAACCCTCGTCTTTAGTTACCAGCAAGTAAAGTTGGGGACTCTAGAGATACTGCCGGTGATAAACCGGAGGAAGGTGGGGATGACGTCAAATCATCATGCCCTATATGTCTTGGGCTACACACGTGCTACAATGGTCTGAACAAAGCGCAGCGAACTCGTGAGGGTAAGCAAATCGCAGAAAACAGATCTCAGTTCGGATTGTAGGCTGCAACTCGCCTACATGAAGTCGGAGTTGCTAGTAATCGTGGATCAGAATGCCACGGTGAATGCGTTCCCGGGTCTTGTACACACCGCCCGTCACACCATGGGAGTTGGCAATACCCGAAGTCGTCGAGCTAACCTTTTAGGAGGCAGACGCCGAAGGTAGGGTCAATAACTGGGGTGAAGTCGTAACAAGGTAGCCGTATCGGAAGGTGCGGCTGGATC

>gi|265678929|ref|NR_029237.1| Helcococcus kunzii strain 22 16S ribosomal RNA gene, partial sequence

GAGAGTTTGATCCTGGCTCAGGACGAACGCTGGCGGCGTGCTTAACACATGCAAGTTGAACGAGAATTTT

TCAGTTGATTTCTTCGGAATGAAACCGAAAANGGAAAGTAGCGAACGGGTGAGTAACACGTGAGAAACCTGCCTTTCACAAAGGGATAGCCTCGGGAAACCGGGATTAATACCTTATGATACATTAATATCGCATGATGTAAATGATGTAAATGTTAAAGAATTTCGGTGAAAGATGGTCTCGCGTCTGATTAGCTAGTTGGTAAGGTACTGGCTTACCAAGGCAACGATCAGTAGCCGGATTGAGAGGTTGAACGGCCACACTGGAACTGAGACACGGTCCAGACTCCTACGGGAGGCAGCAGTGGGGAATTTTGCACAATGGGGGAAACCCTGATGCAGCGACGCCGCGTGAACGATGAAGGTCTTCGGATTGTAAAGTTCTGTCCTTAGCGAAGATAATGACAGTAGCTAAGAAGCAAGCCCTGGCTAAATACGTGCCAGCAGCCGCGGTAATACGTATGGGGCAAGCGTTGTCCGGAATTATTGGGCGTAAAGGGTANGTAGGCGGTTAATTAAGTCTGAATTTAAAGGCTGTGGCTCAACCACGGTTCGGTTTAGAAAACTGGTTAACTTGAGTAGATGAGGGGAAAGTGGAATTCCATGTGTAGCGGTGAAATGCGTAGATATATGGAGGAACACCAGTGGCGAAGGCGACTTTCTAGAATCTAACTGACGCTGAGATACGAAGGCGTGGGTAGCAAACAGGATTAGATACCCTGGTAGTCCACGCAGTAAACGATGAGTGCTAGTTGTTGGGAGTCAAATCTCAGTGACGCAGCTAACGCATTAAGCACTCCGCCTGGGGAGTACGTACGCAAGTATGAAACTCAAAGGAATTGACGGGGACCCGCACAAGCAGCGGAGATGTGGTTTAATTCGAAGCAACGCGAAGAACCTTACCAAGGCTTGAAGGGANATCCTAGAGATAGGATAGTCTTTTCGGAGACTTGTATACAGGTGGTGCATGGTTGTCGTCAGCTCGTGTCGTGAGATGTTGGGTTAAGTCCCGTAACGAGCGCAACCCCTATCTTTAGTTACTAGCGAGTAAAGTCGAGGACTCTAGAGAGACTGCCGGTGATAAACCGGAGGAAGGTGGGGATGACGTCAAATCATCATGCCCTATATGTCTTGGGCTACACACGTGCTACAATGGTCTGAACAAAGCGTAGCTACCTCGTGAGAGCAAGCGAATCGCATAAAACAGATCTCAGTTCGGATTGCAGGCTGCAACTCGCCTGCATGAAGTCGGAGTTGCTAGTAATCGTGGATCAGAACGCCACGGAGAATGCGTTCCCGGGTCTTGTACACACCGCCCGTCACACCATGGGAGTTGGCAATACCCGAAGTCGTCGAGCTAACCGTCAAGGAGGCAGACGCCGAAGGTTGGGTAGAT

>gi|45550016|gb|AY559040.1| Helcococcus pyogenica 16S ribosomal RNA gene, partial sequence

AGAGTTTGATCCTGGCTCAGGACGAACGCTGGCGGCGTGCTTAACACATGCAAGTTGAACGTGATTTTAT

AGACGGATTTCTTCGGAATGACGATTATAAATGAAAGTAGCGAACGGGTGAGTAACACGTGAGAAACCTGCCTTTCACAAAGGGATAGCCTCGGGAAACCGGGATTAATACCTTATGATACATAGAAATCGCATGATAGCTATGTTAAAGATTTTATCGGTGAAAGATGGTCTCGCGTCTGATTAGCTAGTTGGTGGGGTAAAGGCCTACCAAGGCGATGATCAGTAGCCGGATTGAGAGGTTGAACGGCCACACTGGAACTGAGACACGGTCCCAGACTCCTACGGGAGGCAGCAGTGGGGAATTTTGCACAATGGGGGAAACCCTGATGCAGCGACGCCGCGTGTACGATGAAGATCTTCGGATTGTAAAGTACTGTCCTTGGTGAAGATAATGACTGTAACCAAGAAGCAAGCCCTGGCTAAATACGTGCCAGCAGCCGCGGTAA

Part three：other *H. ovis*

>gi|224581446|ref|NR_027228.1| Helcococcus ovis strain s840-96-2 16S ribosomal RNA gene, partial sequence

TGGCTCAGGACGAACGCTGGCGGCGTGCTTAACACATGCAAGTTGAACGAGAATTTTTTAATTAATTTCT

TCGGGAAGAGATTAAAGAAGGAAAGTAGCGAACGGGTGAGTAACACGTGAGAAACCTGCCTTTCACAAAGGGATAGCCTCGGGAAACCGGGATTAATACCTTATGACACTTAGATATCGCATGATAATTAAGTTAAAGAATTTCGGTGAAAGATGGTCTCGCGTCTGATTAGCTAGTTGGTAAGGTAACGGCTTACCAAGGCGACGATCAGTAGCCGGATTGAGAGGTTGAACGGCCACACTGGAACTGAGACACGGTCCAGACTCCTACGGGAGGCAGCAGTGGGGAATTTTGCACAATGGGGGGAACCCTGATGCAGCGACGCCGCGTGAACGATGAAGGTCTTCGGATTGTAAAGTTCTGTCCTTAGTGAAGATAATGACTGTAACTAAGAAGCAAGCCCTGGCTAAATACGTGCCAGCAGCCGCGGTAATACGTATGGGGCAAGCGTTGTCCGGAATTATTGGGCGTAAAGGGTACGTAGGCGGTAATTTAAGTCTGAATTTAAAGGCTGTGGCTCAACCATAGTAAGGTTCAGATACTGGATTACTTGAGTAGATGAGGGGAAAGTGGAATTCCATGTGTAGCGGTGAAATGCGTAGATATATGGAGGAACACCTGTGGCGAAGGCGACTTTCTGGAATCTAACTGACGCTGAGGTACGAAGGCGTGGGGAGCAAACAGGATTAGATACCCTGGTAGTCCACGCAGTAAACGATGAGTGCTAGTTGTCGGGAGTCAAATCTCGGTGACGCAGCTAACGCATTAAGCACTCCGCCTGGGGAGTACGTACGCAAGTATGAAACTCAAAGGAATTGACGGGGACCCGCACAAGCAGCGGAGCATGTGGTTTAATTCGAAGCAACGCGAAGAACCTTACCAAGGCTTGACATATACAGGGATATACTAGAGATAGTATAGTTTCTTCGGAAACTTGTATACAGGTGGTGCATGGTTGTCGTCAGCTCGTGTCGTGAGATGTTGGGTTAAGTCCCGTAACGAGCGCAACCCTTATCTTTAGTTACCAGCATTTCGGATGGGGACTCTAGAGAGACTGCCGGTGATAAACCGGAGGAAGGTGGGGATGACGTCAAATCATCATGCCCTATATGTCTTGGGCTACACACGTGCTACAATGGTCTGAACAAAGCGCAGCTACCTCGTGAGAGCAAGCGAATCGCATAAAACAGATCTCAGTTCGGATTGTAGGCTGCAACTCGCCTACATGAAGTCGGAGTTGCTAGTAATCGTGGATCAGAACGCCACGGTGAATGCGTTCCCGGGTCTTGTACACACCGCCCGTCACACCATGGGAGTTGGCAATACCCGAAGCCGTCGAGCTAACCGTTAGGA

>gi|658157412|gb|KJ676644.1| Helcococcus ovis strain YYQ1403 16S ribosomal RNA gene, partial sequence

ATACCTTATGACACTTAGATATCGCATGATAATTAAGTTAAAGAATTTCGGTGAAAGATGGTCTCGCGTC

TGATTAGCTAGTTGGTAAGGTAACGGCTTACCAAGGCGACGATCAGTAGCCGGATTGAGAGGTTGAACGGCCACACTGGAACTGAGACACGGTCCAGACTCCTACGGGAGGCAGCAGTGGGGAATTTTGCACAATGGGGGGAACCCTGATGCAGCGACGCCGCGTGAACGATGAAGGTCTTCGGATTGTAAAGTTCTGTCCTTAGTGAAGATAATGACTGTAACTAAGAAGCAAGCCCTGGCTAAATACGTGCCAGCAGCCGCGGTAATACGTATGGGGCAAGCGTTGTCCGGAATTATTGGGCGTAAAGGGTACGTAGGCGGTAATTTAAGTCTGAATTTAAAGGCTGTGGCTCAACCATAGTAAGGTTCAGATACTGGATTACTTGAGTAGATGAGGGGAAAGTGGAATTCCATGTGTAGCGGTGAAATGCGTAGATATATGGAGGAACACCTGTGGCGAAGGCGACTTTCTGGAATCTAACTGACGCTGAGGTACGAAGGCGTGGGGAGCAAACAGGATTAGATACCCTGGTAGTCCACGCAGTAAACGATGAGTGCTAGTTGTCGGGAGTCAAATCTCGGTGACGCAGCTAACGCATTAAGCACTCCGCCTGGGGAGTACGTACGCAAGTATGAAACTCAAAGGAATTGACGGGGACCCGCACAAGCAGCGGAGCATGTGGTTTAATTCGAAGCAACGCGAAGAACCTTACCAAGGCTTGACATATACAGG

>gi|384040681|gb|JN861733.1| Helcococcus ovis strain 1105 16S ribosomal RNA gene, partial sequence

GGCGGTAATTTAAGTCTGAATTTAAAGGCTGTGGCTCAACCATAGTAAGGTTCAGATACTGGATTACTTG

AGTAGATGAGGGGAAAGTGGAATTCCATGTGTAGCGGTGAAATGCGTAGATATATGGAGGAACACCTGTGGCGAAGGCGACTTTCTGGAATCTAACTGACGCTGAGGTACGAAGGCGTGGGGAGCACACAGGATTAGATACCCTGGTAGTCCACGCAGTAAACGATGAGTGCTAGTTGTCGGGAGTCAAATCTCGGTGACGCAGCTAACGCATTAAGCACTCCGCCTGGGGAGTACGTACGCAAGTATGAAACTCAAAGGAATTGACGGGGACCCGCACAAGCAGCGGAGCATGTGGTTTAATTCGAAGCAACGCGAAGAACCTTACCAAGGCTTGACATATACAGGGATATACTAGAGATAGTATAGTTTCTTCGGAAACTTGTATACAGGTGGTGCATGGTTGTCGTCAGCTCGTGTCGTGAGATGTTGGGTTAAGTCCCGTAACGAGCGCAACCCTTATCTTTAGTTACCAGCATTTCGGATGGGGACTCTAGAGAGACTGCCGGTGATAAACCGGAGGAAGGTGGGGATGACGTCAAATCATCATGCCCTATATGTCTTGGGCTACACACGTGCTACAATGGTCTGAACAAAGCGCAGCTACCTCGTGAGAGCAAGCGAATCGCATAAAACAGATCTCAGTTCGGATTGTAGGCTGCAACTCGCCTACATGAAGTCGGAGTTGCTAGTAATCGTGGATCAGAACGCCACGGTGAATGCGTTCCCGGGTCTTGTACACACCGCCCGTCACACCATGGGAGTTGGCAATACCCGAAGCCGTCGAGCTAACCGTTAGGA

>gi|284049430|dbj|AB542090.1| Helcococcus ovis gene for 16S ribosomal RNA, partial sequence, strain: H29-Yamagata-000919

TGCTTAACACATGCAAGTTGAACGAGAATTTTTTAATTAATTTCTTCGGAAAGAGATTAAAGAAGGAAAG

TAGCGAACGGGTGAGTAACACGTGAGAAACCTGCCTTTCACAAAGGGATAGCCTCGGGAAACCGGGATTAATACCTTATGACACTTAGATATCGCATGATAATTAAGTTAAAGAATTTCGGTGAAAGATGGTCTCGCGTCTGATTAGCTAGTTGGTAAGGTAACGGCTTACCAAGGCGACGATCAGTAGCCGGATTGAGAGGTTGAACGGCCACACTGGAACTGAGACACGGTCCAGACTCCTACGGGAGGCAGCAGTGGGGAATTTTGCACAATGGGGGGAACCCTGATGCAGCGACGCTGCGTGAACGATGAAGGTCTTCGGATTGTAAAGTTCTGTCCTTAGTGAAGATAATGACTGTAACTAAGAAGCAAGCCCTGGCTAAATACGTGCCAGCAGCCGCGGTAATACGTATGGGGCAAGCGTTGTCCGGAATTATTGGGCGTAAAGGGTACGTAGGCGGTAATTTAAGTCTGAATTTAAAGGCTGTGGCTCAACCATAGTAAGGTTCAGATACTGGATTACTTGAGTAGATGAGGGGAAAGTGGAATTCCATGTGTAGCGGTGAAATGCGTAGATATATGGAGGAACACCTGTGGCGAAGGCGACTTTCTGGAATCTAACTGACGCTGAGGTACGAAGGCGTGGGGAGCAAACAGGATTAGATACCCTGGTAGTCCACGCAGTAAACGATGAGTGCTAGTTGTCGGGAGTCAAATCTCGGTGACGCAGCTAACGCATTAAGCACTCCGCCTGGGGAGTACGTACGCAAGTATGAAACTCAAAGGAATTGACGGGGACCCGCACAAGCAGCGGAGCATGTGGTTTAATTCGAAGCAACGCGAAGAACCTTACCAAGGCTTGACATATACAGGGATATACTAGAGATAGTATAGTTTCTTCGGAAACTTGTATACAGGTGGTGCATGGTTGTCGTCAGCTCGTGTCGTGAGATGTTGGGTTAAGTCCCGTAACGAGCGCAACCCTTATCTTTAGTTACCAGCATTTTGGATGGGGACTCTAGAGAGACTGCCGGTGATAAACCGGAGGAAGGTGGGGATGACGTCAAATCATCATGCCCTATATGTCTTGGGCTACACACGTGCTACAATGGTCTGAACAAAGCGCAGCTACCTCGTGAGAGCAAGCGAATCGCATAAAACAGATCTCAGTTCGGATTGTAGGCTGCAACTCGCCTACATGAAGTCGGAGTTGCTAGTAATCGTGGATCAGAACGCCACGGTGAATGCGTTCCCGGGTCTTGTACACACCGCCCGTCACACCATGGGAGTTGGCAATACCCGAAGCCGTCGAGCTAACCGTTAGGA

>gi|284049429|dbj|AB542089.1| Helcococcus ovis gene for 16S ribosomal RNA, partial sequence, strain: H21-Yamagata-000808

TGCTTAACACATGCAAGTTGAACGAGAATTTTTTAATTAATTTCTTCGGAAAGAGATTAAAGAAGGAAAG

TAGCGAACGGGTGAGTAACACGTGAGAAACCTGCCTTTCACAAAGGGATAGCCTCGGGAAACCGGGATTAATACCTTATGACACTTAGATATCGCATGATAATTAAGTTAAAGAATTTCGGTGAAAGATGGTCTCGCGTCTGATTAGCTAGTTGGTAAGGTAACGGCTTACCAAGGCGACGATCAGTAGCCGGATTGAGAGGTTGAACGGCCACACTGGAACTGAGACACGGTCCAGACTCCTACGGGAGGCAGCAGTGGGGAATTTTGCACAATGGGGGGAACCCTGATGCAGCGACGCCGCGTGAACGATGAAGGTCTTCGGATTGTAAAGTTCTGTCCTTAGTGAAGATAATGACTGTAACTAAGAAGCAAGCCCTGGCTAAATACGTGCCAGCAGCCGCGGTAATACGTATGGGGCAAGCGTTGTCCGGAATTATTGGGCGTAAAGGGTACGTAGGCGGTAATTTAAGTCTGAATTTAAAGGCTGTGGCTCAACCATAGTAAGGTTCAGATACTGGATTACTTGAGTAGATGAGGGGAAAGTGGAATTCCATGTGTAGCGGTGAAATGCGTAGATATATGGAGGAACACCTGTGGCGAAGGCGACTTTCTGGAATCTAACTGACGCTGAGGTACGAAGGCGTGGGGAGCAAACAGGATTAGATACCCTGGTAGTCCACGCAGTAAACGATGAGTGCTAGTTGTCGGGAGTCAAATCTCGGTGACGCAGCTAACGCATTAAGCACTCCGCCTGGGGAGTACGTACGCAAGTATGAAACTCAAAGGAATTGACGGGGACCCGCACAAGCAGCGGAGCATGTGGTTTAATTCGAAGCAACGCGAAGAACCTTACCAAGGCTTGACATATACAGGGATATACTAGAGATAGTATAGTTTCTTCGGAAACTTGTATACAGGTGGTGCATGGTTGTCGTCAGCTCGTGTCGTGAGATGTTGGGTTAAGTCCCGTAACGAGCGCAACCCTTATCTTTAGTTACCAGCATTTTGGATGGGGACTCTAGAGAGACTGCCGGTGATAAACCGGAGGAAGGTGGGGATGACGTCAAATCATCATGCCCTATATGTCTTGGGCTACACACGTGCTACAATGGTCTGAACAAAGCGCAGCTACCTCGCGAGAGCAAGCGAATCGCATAAAACAGATCTCAGTTCGGATTGTAGGCTGCAACTCGCCTACATGAAGTCGGAGTTGCTAGTAATCGTGGATCAGAACGCCACGGTGAATGCGTTCCCGGGTCTTGTACACACCGCCCGTCACACCATGGGAGTTGGCAATACCCGAAGCCGTCGAGCTAACCGTTAGGA

>gi|284049428|dbj|AB542088.1| Helcococcus ovis gene for 16S ribosomal RNA, partial sequence, strain: H14-Yamagata-000307

TGCTTAACACATGCAAGTTGAACGAGAATTTTTTAATTAATTTCTTCGGAAAGAGATTAAAGAAGGAAAG

TAGCGAACGGGTGAGTAACACGTGAGAAACCTGCCTTTCACAAAGGGATAGCCTCGGGAAACCGGGATTAATACCTTATGACACTTAGATATCGCATGATAATTAAGTTAAAGAATTTCGGTGAAAGATGGTCTCGCGTCTGATTAGCTAGTTGGTAAGGTAACGGCTTACCAAGGCGACGATCAGTAGCCGGATTGAGAGGTTGAACGGCCACACTGGAACTGAGACACGGTCCAGACTCCTACGGGAGGCAGCAGTGGGGAATTTTGCACAATGGGGGGAACCCTGATGCAGCGACGCCGCGTGAACGATGAAGGTCTTCGGATTGTAAAGTTCTGTCCTTAGTGAAGATAATGACTGTAACTAAGAAGCAAGCCCTGGCTAAATACGTGCCAGCAGCCGCGGTAATACGTATGGGGCAAGCGTTGTCCGGAATTATTGGGCGTAAAGGGTACGTAGGCGGTAATTTAAGTCTGAATTTAAAGGCTGTGGCTCAACCATAGTAAGGTTCAGATACTGGATTACTTGAGTAGATGAGGGGGAAGTGGAATTCCATGTGTAGCGGTGAAATGCGTAGATATATGGAGGAACACCTGTGGCGAAGGCGACTTTCTGGAATCTAACTGACGCTGAGGTACGAAGGCGTGGGGAGCAAACAGGATTAGATACCCTGGTAGTCCACGCAGTAAACGATGAGTGCTAGTTGTCGGGAGTCAAATCTCGGTGACGCAGCTAACGCATTAAGCACTCCGCCTGGGGAGTACGTACGCAAGTATGAAACTCAAAGGAATTGACGGGGACCCGCACAAGCAGCGGAGCATGTGGTTTAATTCGAAGCAACGCGAAGAACCTTACCAAGGCTTGACATATACAGGGATATACTAGAGATAGTATAGTTTCTTCGGAAACTTGTATACAGGTGGTGCATGGTTGTCGTCAGCTCGTGTCGTGAGATGTTGGGTTAAGTCCCGTAACGAGCGCAACCCTTATCTTTAGTTACCAGCATTTCGGATGGGGACTCTAGAGAGACTGCCGGTGATAAACCGGAGGAAGGTGGGGATGACGTCAAATCATCATGCCCTATATGTCTTGGGCTACACACGTGCTACAATGGTCTGAACAAAGCGCAGCTACCTCGTGAGAGCAAGCGAATCGCATAAAACAGATCTCAGTTCGGATTGTAGGCTGCAACTCGCCTACATGAAGTCGGAGTTGCTAGTAATCGTGGATCAGAACGCCACGGTGAATGCGTTCCCGGGTCTTGTACACACCGCCCGTCACACCATGGGAGTTGGCAATACCCGAAGCCGTCGAGCTAACCGTTAGGA

>gi|284049427|dbj|AB542087.1| Helcococcus ovis gene for 16S ribosomal RNA, partial sequence, strain: H41-Yamagata-080523

TGCTTAACACATGCAAGTTGAACGAGAATTTTTTAATTAATTTCTTCGGAAAGAGATTAAAGAAGGAAAG

TAGCGAACGGGTGAGTAACACGTGAGAAACCTGCCTTTCACAAAGGGATAGCCTCGGGAAACCGGGATTAATACCTTATGACACTTAGATATCGCATGATAATTAAGTTAAAGAATTTCGGTGAAAGATGGTCTCGCGTCTGATTAGCTAGTTGGTAAGGTAACGGCTTACCAAGGCGACGATCAGTAGCCGGATTGAGAGGTTGAACGGCCACACTGGAACTGAGACACGGTCCAGACTCCTACGGGAGGCAGCAGTGGGGAATTTTGCACAATGGGGGGAACCCTGATGCAGCGACGCCGCGTGAACGATGAAGGTCTTCGGATTGTAAAGTTCTGTCCTTAGTGAAGATAATGACTGTAACTAAGAAGCAAGCCCTGGCTAAATACGTGCCAGCAGCCGCGGTAATACGTATGGGGCAAGCGTTGTCCGGAATTATTGGGCGTAAAGGGTACGTAGGCGGTAATTTAAGTCTGAATTTAAAGGCTGTGGCTCAACCATAGTAAGGTTCAGATACTGGATTACTTGAGTAGATGAGGGGAAAGTGGAATTCCATGTGTAGCGGTGAAATGCGTAGATATATGGAGGAACACCTGTGGCGAAGGCGACTTTCTGGAATCTAACTGACGCTGAGGTACGAAGGCGTGGGGAGCAAACAGGATTAGATACCCTGGTAGTCCACGCAGTAAACGATGAGTGCTAGTTGTCGGGAGTCAAATCTCGGTGACGCAGCTAACGCATTAAGCACTCCGCCTGGGGAGTACGTACGCAAGTATGAAACTCAAAGGAATTGACGGGGACCCGCACAAGCAGCGGAGCATGTGGTTTAATTCGAAGCAACGCGAAGAACCTTACCAAGGCTTGACATATACAGGGATATACTAGAGATAGTATAGTTTCTTCGGAAACTTGTATACAGGTGGTGCATGGTTGTCGTCAGCTCGTGTCGTGAGATGTTGGGTTAAGTCCCGTAACGAGCGCAACCCTTATCTTTAGTTACCAGCATTTCGGATGGGGACTCTAGAGAGACTGCCGGTGATAAACCGGAGGAAGGTGGGGATGACGTCAAATCATCATGCCCTATATGTCTTGGGCTACACACGTGCTACAATGGTCTGAACAAAGCGCAGCTACCTCGTGAGAGCAAGCGAATCGCATAAAACAGATCTCAGTTCGGATTGTAGGCTGCAACTCGCCTACATGAAGTCGGAGTTGCTAGTAATCGTGGATCAGAACGCCACGGTGAATGCGTTCCCGGGTCTTGTACACACCGCCCGTCACACCATGGGAGTTGGCAATACCCGAAGCCGTCGAGCTAACCGTTAGGA

>gi|284049426|dbj|AB542086.1| Helcococcus ovis gene for 16S ribosomal RNA, partial sequence, strain: H37-Yamagata-080312

TGCTTAACACATGCAAGTTGAACGAGAATTTTTTAATTAATTTCTTCGGAAAGAGATTAAAGAAGGAAAG

TAGCGAACGGGTGAGTAACACGTGAGAAACCTGCCTTTCACAAAGGGATAGCCTCGGGAAACCGGGATTAATACCTTATGACACTTAGATATCGCATGATAATTAAGTTAAAGAATTTCGGTGAAAGATGGTCTCGCGTCTGATTAGCTAGTTGGTAAGGTAACGGCTTACCAAGGCGACGATCAGTAGCCGGATTGAGAGGTTGAACGGCCACACTGGAACTGAGACACGGTCCAGACTCCTACGGGAGGCAGCAGTGGGGAATTTTGCACAATGGGGGGAACCCTGATGCAGCGACGCCGCGTGAACGATGAAGGTCTTCGGATTGTAAAGTTCTGTCCTTAGTGAAGATAATGACTGTAACTAAGAAGCAAGCCCTGGCTAAATACGTGCCAGCAGCCGCGGTAATACGTATGGGGCAAGCGTTGTCCGGAATTATTGGGCGTAAAGGGTACGTAGGCGGTAATTTAAGTCTGAATTTAAAGGCTGTGGCTCAACCATAGTAAGGTTCAGATACTGGATTACTTGAGTAGATGAGGGGAAAGTGGAATTCCATGTGTAGCGGTGAAATGCGTAGATATATGGAGGAACACCTGTGGCGAAGGCGACTTTCTGGAATCTAACTGACGCTGAGGTACGAAGGCGTGGGGAGCAAACAGGATTAGATACCCTGGTAGTCCACGCAGTAAACGATGAGTGCTAGTTGTCGGGAGTCAAATCTCGGTGACGCAGCTAACGCATTAAGCACTCCGCCTGGGGAGTACGTACGCAAGTATGAAACTCAAAGGAATTGACGGGGACCCGCACAAGCAGCGGAGCATGTGGTTTAATTCGAAGCAACGCGAAGAACCTTACCAAGGCTTGACATATACAGGGATATACTAGAGATAGTATAGTTTCTTCGGAAACTTGTATACAGGTGGTGCATGGTTGTCGTCAGCTCGTGTCGTGAGATGTTGGGTTAAGTCCCGTAACGAGCGCAACCCTTATCTTTAGTTACCAGCATTTCGGATGGGGACTCTAGAGAGACTGCCGGTGATAAACCGGAGGAAGGTGGGGATGACGTCAAATCATCATGCCCTATATGTCTTGGGCTACACACGTGCTACAATGGTCTGAACAAAGCGCAGCTACCTCGTGAGAGCAAGCGAATCGCATAAAACAGATCTCAGTTCGGATTGTAGGCTGCAACTCGCCTACATGAAGTCGGAGTTGCTAGTAATCGTGGATCAGAACGCCACGGTGAATGCGTTCCCGGGTCTTGTACACACCGCCCGTCACACCATGGGAGTTGGCAATACCCGAAGCCGTCGAGCTAACCGTTAGGA

>gi|284049425|dbj|AB542085.1| Helcococcus ovis gene for 16S ribosomal RNA, partial sequence, strain: H34-Yamagata-970220

TGCTTAACACATGCAAGTTGAACGAGAATTTTTTAATTAATTTCTTCGGAAAGAGATTAAAGAAGGAAAG

TAGCGAACGGGTGAGTAACACGTGAGAAACCTGCCTTTCACAAAGGGATAGCCTCGGGAAACCGGGATTAATACCTTATGACACTTAGATATCGCATGATAATTAAGTTAAAGAATTTCGGTGAAAGATGGTCTCGCGTCTGATTAGCTAGTTGGTAAGGTAACGGCTTACCAAGGCGACGATCAGTAGCCGGATTGAGAGGTTGAACGGCCACACTGGAACTGAGACACGGTCCAGACTCCTACGGGAGGCAGCAGTGGGGAATTTTGCACAATGGGGGGAACCCTGATGCAGCGACGCCGCGTGAACGATGAAGGTCTTCGGATTGTAAAGTTCTGTCCTTAGTGAAGATAATGACTGTAACTAAGAAGCAAGCCCTGGCTAAATACGTGCCAGCAGCCGCGGTAATACGTATGGGGCAAGCGTTGTCCGGAATTATTGGGCGTAAAGGGTACGTAGGCGGTAATTTAAGTCTGAATTTAAAGGCTGTGGCTCAACCATAGTAAGGTTCAGATACTGGATTACTTGAGTAGATGAGGGGAAAGTGGAATTCCATGTGTAGCGGTGAAATGCGTAGATATATGGAGGAACACCTGTGGCGAAGGCGACTTTCTGGAATCTAACTGACGCTGAGGTACGAAGGCGTGGGGAGCAAACAGGATTAGATACCCTGGTAGTCCACGCAGTAAACGATGAGTGCTAGTTGTCGGGAGTCAAATCTCGGTGACGCAGCTAACGCATTAAGCACTCCGCCTGGGGAGTACGTACGCAAGTATGAAACTCAAAGGAATTGACGGGGACCCGCACAAGCAGCGGAGCATGTGGTTTAATTCGAAGCAACGCGAAGAACCTTACCAAGGCTTGACATATACAGGGATATACTAGAGATAGTATAGTTTCTTCGGAAACTTGTATACAGGTGGTGCATGGTTGTCGTCAGCTCGTGTCGTGAGATGTTGGGTTAAGTCCCGTAACGAGCGCAACCCTTATCTTTAGTTACCAGCATTTCGGATGGGGACTCTAGAGAGACTGCCGGTGATAAACCGGAGGAAGGTGGGGATGACGTCAAATCATCATGCCCTATATGTCTTGGGCTACACACGTGCTACAATGGTCTGAACAAAGCGCAGCTACCTCGTGAGAGCAAGCGAATCGCATAAAACAGATCTCAGTTCGGATTGTAGGCTGCAACTCGCCTACATGAAGTCGGAGTTGCTAGTAATCGTGGATCAGAACGCCACGGTGAATGCGTTCCCGGGTCTTGTACACACCGCCCGTCACACCATGGGAGTTGGCAATACCCGAAGCCGTCGAGCTAACCGTTAGGA

>gi|284049423|dbj|AB542083.1| Helcococcus ovis gene for 16S ribosomal RNA, partial sequence, strain: H12-Yamagata-991222

TGCTTAACACATGCAAGTTGAACGAGAATTTTTTAATTAATTTCTTCGGAAAGAGATTAAAGAAGGAAAG

TAGCGAACGGGTGAGTAACACGTGAGAAACCTGCCTTTCACAAAGGGATAGCCTCGGGAAACCGGGATTAATACCTTATGACACTTAGATATCGCATGATAATTAAGTTAAAGAATTTCGGTGAAAGATGGTCTCGCGTCTGATTAGCTAGTTGGTAAGGTAACGGCTTACCAAGGCGACGATCAGTAGCCGGATTGAGAGGTTGAACGGCCACACTGGAACTGAGACACGGTCCAGACTCCTACGGGAGGCAGCAGTGGGGAATTTTGCACAATGGGGGGAACCCTGATGCAGCGACGCCGCGTGAACGATGAAGGTCTTCGGATTGTAAAGTTCTGTCCTTAGTGAAGATAATGACTGTAACTAAGAAGCAAGCCCTGGCTAAATACGTGCCAGCAGCCGCGGTAATACGTATGGGGCAAGCGTTGTCCGGAATTATTGGGCGTAAAGGGTACGTAGGCGGTAATTTAAGTCTGAATTTAAAGGCTGTGGCTCAACCATAGTAAGGTTCAGATACTGGATTACTTGAGTAGATGAGGGGAAAGTGGAATTCCATGTGTAGCGGTGAAATGCGTAGATATATGGAGGAACACCTGTGGCGAAGGCGACTTTCTGGAATCTAACTGACGCTGAGGTACGAAGGCGTGGGGAGCAAACAGGATTAGATACCCTGGTAGTCCACGCAGTAAACGATGAGTGCTAGTTGTCGGGAGTCAAATCTCGGTGACGCAGCTAACGCATTAAGCACTCCGCCTGGGGAGTACGTACGCAAGTATGAAACTCAAAGGAATTGACGGGGACCCGCACAAGCAGCGGAGCATGTGGTTTAATTCGAAGCAACGCGAAGAACCTTACCAAGGCTTGACATATACAGGGATATACTAGAGATAGTATAGTTTCTTCGGAAACTTGTATACAGGTGGTGCATGGTTGTCGTCAGCTCGTGTCGTGAGATGTTGGGTTAAGTCCCGTAACGAGCGCAACCCTTATCTTTAGTTACCAGCATTTCGGATGGGGACTCTAGAGAGACTGCCGGTGATAAACCGGAGGAAGGTGGGGATGACGTCAAATCATCATGCCCTATATGTCTTGGGCTACACACGTGCTACAATGGTCTGAACAAAGCGCAGCTACCTCGTGAGAGCAAGCGAATCGCATAAAACAGATCTCAGTTCGGATTGTAGGCTGCAACTCGCCTACATGAAGTCGGAGTTGCTAGTAATCGTGGATCAGAACGCCACGGTGAATGCGTTCCCGGGTCTTGTACACACCGCCCGTCACACCATGGGAGTTGGCAATACCCGAAGCCGTCGAGCTAACCGTTAGGA

>gi|284049422|dbj|AB542082.1| Helcococcus ovis gene for 16S ribosomal RNA, partial sequence, strain: H11-Yamagata-991124

TGCTTAACACATGCAAGTTGAACGAGAATTTTTTAATTAATTTCTTCGGAAAGAGATTAAAGAAGGAAAG

TAGCGAACGGGTGAGTAACACGTGAGAAACCTGCCTTTCACAAAGGGATAGCCTCGGGAAACCGGGATTAATACCTTATGACACTTAGATATCGCATGATAATTAAGTTAAAGAATTTCGGTGAAAGATGGTCTCGCGTCTGATTAGCTAGTTGGTAAGGTAACGGCTTACCAAGGCGACGATCAGTAGCCGGATTGAGAGGTTGAACGGCCACACTGGAACTGAGACACGGTCCAGACTCCTACGGGAGGCAGCAGTGGGGAATTTTGCACAATGGGGGGAACCCTGATGCAGCGACGCCGCGTGAACGATGAAGGTCTTCGGATTGTAAAGTTCTGTCCTTAGTGAAGATAATGACTGTAACTAAGAAGCAAGCCCTGGCTAAATACGTGCCAGCAGCCGCGGTAATACGTATGGGGCAAGCGTTGTCCGGAATTATTGGGCGTAAAGGGTACGTAGGCGGTAATTTAAGTCTGAATTTAAAGGCTGTGGCTCAACCATAGTAAGGTTCAGATACTGGATTACTTGAGTAGATGAGGGGAAAGTGGAATTCCATGTGTAGCGGTGAAATGCGTAGATATATGGAGGAACACCTGTGGCGAAGGCGACTTTCTGGAATCTAACTGACGCTGAGGTACGAAGGCGTGGGGAGCAAACAGGATTAGATACCCTGGTAGTCCACGCAGTAAACGATGAGTGCTAGTTGTCGGGAGTCAAATCTCGGTGACGCAGCTAACGCATTAAGCACTCCGCCTGGGGAGTACGTACGCAAGTATGAAACTCAAAGGAATTGACGGGGACCCGCACAAGCAGCGGAGCATGTGGTTTAATTCGAAGCAACGCGAAGAACCTTACCAAGGCTTGACATATACAGGGATATACTAGAGATAGTATAGTTTCTTCGGAAACTTGTATACAGGTGGTGCATGGTTGTCGTCAGCTCGTGTCGTGAGATGTTGGGTTAAGTCCCGTAACGAGCGCAACCCTTATCTTTAGTTACCAGCATTTCGGATGGGGACTCTAGAGAGACTGCCGGTGATAAACCGGAGGAAGGTGGGGATGACGTCAAATCATCATGCCCTATATGTCTTGGGCTACACACGTGCTACAATGGTCTGAACAAAGCGCAGCTACCTCGTGAGAGCAAGCGAATCGCATAAAACAGATCTCAGTTCGGATTGTAGGCTGCAACTCGCCTACATGAAGTCGGAGTTGCTAGTAATCGTGGATCAGAACGCCACGGTGAATGCGTTCCCGGGTCTTGTACACACCGCCCGTCACACCATGGGAGTTGGCAATACCCGAAGCCGTCGAGCTAACCGTTAGGA

>gi|284049421|dbj|AB542081.1| Helcococcus ovis gene for 16S ribosomal RNA, partial sequence, strain: H10-Yamagata-990517

TGCTTAACACATGCAAGTTGAACGAGAATTTTTTAATTAATTTCTTCGGAAAGAGATTAAAGAAGGAAAG

TAGCGAACGGGTGAGTAACACGTGAGAAACCTGCCTTTCACAAAGGGATAGCCTCGGGAAACCGGGATTAATACCTTATGACACTTAGATATCGCATGATAATTAAGTTAAAGAATTTCGGTGAAAGATGGTCTCGCGTCTGATTAGCTAGTTGGTAAGGTAACGGCTTACCAAGGCGACGATCAGTAGCCGGATTGAGAGGTTGAACGGCCACACTGGAACTGAGACACGGTCCAGACTCCTACGGGAGGCAGCAGTGGGGAATTTTGCACAATGGGGGGAACCCTGATGCAGCGACGCCGCGTGAACGATGAAGGTCTTCGGATTGTAAAGTTCTGTCCTTAGTGAAGATAATGACTGTAACTAAGAAGCAAGCCCTGGCTAAATACGTGCCAGCAGCCGCGGTAATACGTATGGGGCAAGCGTTGTCCGGAATTATTGGGCGTAAAGGGTACGTAGGCGGTAATTTAAGTCTGAATTTAAAGGCTGTGGCTCAACCATAGTAAGGTTCAGATACTGGATTACTTGAGTAGATGAGGGGAAAGTGGAATTCCATGTGTAGCGGTGAAATGCGTAGATATATGGAGGAACACCTGTGGCGAAGGCGACTTTCTGGAATCTAACTGACGCTGAGGTACGAAGGCGTGGGGAGCAAACAGGATTAGATACCCTGGTAGTCCACGCAGTAAACGATGAGTGCTAGTTGTCGGGAGTCAAATCTCGGTGACGCAGCTAACGCATTAAGCACTCCGCCTGGGGAGTACGTACGCAAGTATGAAACTCAAAGGAATTGACGGGGACCCGCACAAGCAGCGGAGCATGTGGTTTAATTCGAAGCAACGCGAAGAACCTTACCAAGGCTTGACATATACAGGGATATACTAGAGATAGTATAGTTTCTTCGGAAACTTGTATACAGGTGGTGCATGGTTGTCGTCAGCTCGTGTCGTGAGATGTTGGGTTAAGTCCCGTAACGAGCGCAACCCTTATCTTTAGTTACCAGCATTTCGGATGGGGACTCTAGAGAGACTGCCGGTGATAAACCGGAGGAAGGTGGGGATGACGTCAAATCATCATGCCCTATATGTCTTGGGCTACACACGTGCTACAATGGTCTGAACAAAGCGCAGCTACCTCGTGAGAGCAAGCGAATCGCATAAAACAGATCTCAGTTCGGATTGTAGGCTGCAACTCGCCTACATGAAGTCGGAGTTGCTAGTAATCGTGGATCAGAACGCCACGGTGAATGCGTTCCCGGGTCTTGTACACACCGCCCGTCACACCATGGGAGTTGGCAATACCCGAAGCCGTCGAGCTAACCGTTAGGA

>gi|284049420|dbj|AB542080.1| Helcococcus ovis gene for 16S ribosomal RNA, partial sequence, strain: H20-Yamagata-000412

TGCTTAACACATGCAAGTTGAACGAGAATTTTTTAATTAATTTCTTCGGAAAGAGATTAAAGAAGGAAAG

TAGCGAACGGGTGAGTAACACGTGAGAAACCTGCCTTTCACAAAGGGATAGCCTCGGGAAACCGGGATTAATACCTTATGACACTTAGATATCGCATGATAATTAAGTTAAAGAATTTCGGTGAAAGATGGTCTCGCGTCTGATTAGCTAGTTGGTAAGGTAACGGCTTACCAAGGCGACGATCAGTAGCCGGATTGAGAGGTTGAACGGCCACACTGGAACTGAGACACGGTCCAGACTCCTACGGGAGGCAGCAGTGGGGAATTTTGCACAATGGGGGAAACCCTGATGCAGCGACGCCGCGTGAACGATGAAGGTCTTCGGATTGTAAAGTTCTGTCCTTAGTGAAGATAATGACTGTAACTAAGAAGCAAGCCCTGGCTAAATACGTGCCAGCAGCCGCGGTAATACGTATGGGGCAAGCGTTGTCCGGAATTATTGGGCGTAAAGGGTACGTAGGCGGTAATTTAAGTCTGAATTTAAAGGCTGTGGCTCAACCATAGTAAGGTTCAGATACTGGATTACTTGAGTAGATGAGGGGAAAGTGGAATTCCATGTGTAGCGGTGAAATGCGTAGATATATGGAGGAACACCTGTGGCGAAGGCGACTTTCTGGAATCTAACTGACGCTGAGGTACGAAGGCGTGGGGAGCAAACAGGATTAGATACCCTGGTAGTCCACGCAGTAAACGATGAGTGCTAGTTGTCGGGAGTCAAATCTCGGTGACGCAGCTAACGCATTAAGCACTCCGCCTGGGGAGTACGTACGCAAGTATGAAACTCAAAGGAATTGACGGGGACCCGCACAAGCAGCGGAGCATGTGGTTTAATTCGAAGCAACGCGAAGAACCTTACCAAGGCTTGACATATACAGGGATATACTAGAGATAGTATAGTTTCTTCGGAAACTTGTATACAGGTGGTGCATGGTTGTCGTCAGCTCGTGTCGTGAGATGTTGGGTTAAGTCCCGTAACGAGCGCAACCCTTATCTTTAGTTACCAGCATTTCGGATGGGGACTCTAGAGAGACTGCCGGTGATAAACCGGAGGAAGGTGGGGATGACGTCAAATCATCATGCCCTATATGTCTTGGGCTACACACGTGCTACAATGGTCTGAACAAAGCGCAGCTACCTCGTGAGAGCAAGCGAATCGCATAAAACAGATCTCAGTTCGGATTGTAGGCTGCAACTCGCCTACATGAAGTCGGAGTTGCTAGTAATCGTGGATCAGAACGCCACGGTGAATGCGTTCCCGGGTCTTGTACACACCGCCCGTCACACCATGGGAGTTGGCAATACCCGAAGCCGTCGAGCTAACCGTTAGGA

>gi|284049419|dbj|AB542079.1| Helcococcus ovis gene for 16S ribosomal RNA, partial sequence, strain: H6-Yamagata-990426

TGCTTAACACATGCAAGTTGAACGAGAATTTTTTAATTAATTTCTTCGGAAAGAGATTAAAGAAGGAAAG

TAGCGAACGGGTGAGTAACACGTGAGAAACCTGCCTTTCACAAAGGGATAGCCTCGGGAAACCGGGATTAATACCTTATGACACTTAGATATCGCATGATAATTAAGTTAAAGAATTTCGGTGAAAGATGGTCTCGCGTCTGATTAGCTAGTTGGTAAGGTAACGGCTTACCAAGGCGACGATCAGTAGCCGGATTGAGAGGTTGAACGGCCACACTGGAACTGAGACACGGTCCAGACTCCTACGGGAGGCAGCAGTGGGGAATTTTGCACAATGGGGGAAACCCTGATGCAGCGACGCCGCGTGAACGATGAAGGTCTTCGGATTGTAAAGTTCTGTCCTTAGTGAAGATAATGACTGTAACTAAGAAGCAAGCCCTGGCTAAATACGTGCCAGCAGCCGCGGTAATACGTATGGGGCAAGCGTTGTCCGGAATTATTGGGCGTAAAGGGTACGTAGGCGGTAATTTAAGTCTGAATTTAAAGGCTGTGGCTCAACCATAGTAAGGTTCAGATACTGGATTACTTGAGTAGATGAGGGGAAAGTGGAATTCCATGTGTAGCGGTGAAATGCGTAGATATATGGAGGAACACCTGTGGCGAAGGCGACTTTCTGGAATCTAACTGACGCTGAGGTACGAAGGCGTGGGGAGCAAACAGGATTAGATACCCTGGTAGTCCACGCAGTAAACGATGAGTGCTAGTTGTCGGGAGTCAAATCTCGGTGACGCAGCTAACGCATTAAGCACTCCGCCTGGGGAGTACGTACGCAAGTATGAAACTCAAAGGAATTGACGGGGACCCGCACAAGCAGCGGAGCATGTGGTTTAATTCGAAGCAACGCGAAGAACCTTACCAAGGCTTGACATATACAGGGATATACTAGAGATAGTATAGTTTCTTCGGAAACTTGTATACAGGTGGTGCATGGTTGTCGTCAGCTCGTGTCGTGAGATGTTGGGTTAAGTCCCGTAACGAGCGCAACCCTTATCTTTAGTTACCAGCATTTCGGATGGGGACTCTAGAGAGACTGCCGGTGATAAACCGGAGGAAGGTGGGGATGACGTCAAATCATCATGCCCTATATGTCTTGGGCTACACACGTGCTACAATGGTCTGAACAAAGCGCAGCTACCTCGTGAGAGCAAGCGAATCGCATAAAACAGATCTCAGTTCGGATTGTAGGCTGCAACTCGCCTACATGAAGTCGGAGTTGCTAGTAATCGTGGATCAGAACGCCACGGTGAATGCGTTCCCGGGTCTTGTACACACCGCCCGTCACACCATGGGAGTTGGCAATACCCGAAGCCGTCGAGCTAACCGTTAGGA

>gi|284049418|dbj|AB542078.1| Helcococcus ovis gene for 16S ribosomal RNA, partial sequence, strain: H1-Yamagata-980922

TGCTTAACACATGCAAGTTGAACGAGAATTTTTTAATTAATTTCTTCGGAAAGAGATTAAAGAAGGAAAG

TAGCGAACGGGTGAGTAACACGTGAGAAACCTGCCTTTCACAAAGGGATAGCCTCGGGAAACCGGGATTAATACCTTATGACACTTAGATATCGCATGATAATTAAGTTAAAGAATTTCGGTGAAAGATGGTCTCGCGTCTGATTAGCTAGTTGGTAAGGTAACGGCTTACCAAGGCGACGATCAGTAGCCGGATTGAGAGGTTGAACGGCCACACTGGAACTGAGACACGGTCCAGACTCCTACGGGAGGCAGCAGTGGGGAATTTTGCACAATGGGGGAAACCCTGATGCAGCGACGCCGCGTGAACGATGAAGGTCTTCGGATTGTAAAGTTCTGTCCTTAGTGAAGATAATGACTGTAACTAAGAAGCAAGCCCTGGCTAAATACGTGCCAGCAGCCGCGGTAATACGTATGGGGCAAGCGTTGTCCGGAATTATTGGGCGTAAAGGGTACGTAGGCGGTAATTTAAGTCTGAATTTAAAGGCTGTGGCTCAACCATAGTAAGGTTCAGATACTGGATTACTTGAGTAGATGAGGGGAAAGTGGAATTCCATGTGTAGCGGTGAAATGCGTAGATATATGGAGGAACACCTGTGGCGAAGGCGACTTTCTGGAATCTAACTGACGCTGAGGTACGAAGGCGTGGGGAGCAAACAGGATTAGATACCCTGGTAGTCCACGCAGTAAACGATGAGTGCTAGTTGTCGGGAGTCAAATCTCGGTGACGCAGCTAACGCATTAAGCACTCCGCCTGGGGAGTACGTACGCAAGTATGAAACTCAAAGGAATTGACGGGGACCCGCACAAGCAGCGGAGCATGTGGTTTAATTCGAAGCAACGCGAAGAACCTTACCAAGGCTTGACATATACAGGGATATACTAGAGATAGTATAGTTTCTTCGGAAACTTGTATACAGGTGGTGCATGGTTGTCGTCAGCTCGTGTCGTGAGATGTTGGGTTAAGTCCCGTAACGAGCGCAACCCTTATCTTTAGTTACCAGCATTTCGGATGGGGACTCTAGAGAGACTGCCGGTGATAAACCGGAGGAAGGTGGGGATGACGTCAAATCATCATGCCCTATATGTCTTGGGCTACACACGTGCTACAATGGTCTGAACAAAGCGCAGCTACCTCGTGAGAGCAAGCGAATCGCATAAAACAGATCTCAGTTCGGATTGTAGGCTGCAACTCGCCTACATGAAGTCGGAGTTGCTAGTAATCGTGGATCAGAACGCCACGGTGAATGCGTTCCCGGGTCTTGTACACACCGCCCGTCACACCATGGGAGTTGGCAATACCCGAAGCCGTCGAGCTAACCGTTAGGA

>gi|635545276|gb|KJ652684.1| Helcococcus ovis strain KH1488C 16S ribosomal RNA gene, partial sequence

GGCTCAGGACGAACGCTGGCGGCGTGCTTAACACATGCAAGTTGAACGAGAAAATTTTGATAGAATCTTC

GGAGGAAAGAGAAATTGGAAAGTAGCGAACGGGTGAGTAACACGTGAGAAACCTGCCTATTACAAAGGGATAGCCTCGGGAAACCGTGATTAATACCTTATGATACATTGTAGTCGCATGACAATGATGTTAAAGTTTTTTCGGTAATAGATGGTCTCGCGTCTGATTAGCTAGATGGTGGGGTAAAGGCCTACCATGGCGACGATCAGTAGCCGGATTGAGAGGTTGAACGGCCACACTGGAACTGAGACACGGTCCAGACTCCTACGGGAGGCAGCAGTGGGGAATTTTGCACAATGGGGGGAACCCTGATGCAGCGACGCCGCGTGAATGAAGAAGGTCTTCGGATTGTAAAATTCTGTCCTTGGTGAAGATAA

>gi|384095088|gb|JQ404450.1| Helcococcus ovis strain XJDY-N1-3 16S ribosomal RNA gene, partial sequence

GTTTAGAGAAGGGAAGTGACGAAAGGGGGAGTAACTGCTGATGAATTGCCTTTCACCTGGGATAGCGGCGGGAAACCGGGATTAATAGCTTATGACGCTTGGAGGGGGTTGGAAAGATATTTTGGAGGATTTCGGTGAAAGATGGTCTCGCGTCGGATTAGCTAGTTGGTAAGGTAACGGCTTACCAAGGCGACGATCAGTAGCCGGATTGAGAGGTTGAACGGCCACACTGGAACTGAGACACGGTCCAGACTCCTACGGGAGGCAGCAGTGGGGAATTTTGCACAATGGGGGAAACCCTGATGCAGCGACGCCGCGTGAACGATGAAGGTCTTCGGATTGTAAAGTTCTGTCCTTAGTGAAGATAATGACTGTAACTAAGAAGCAAGCCCTGGCTAAATACGTGCCAGCAGCCGCGGTAATACGTATGGGGCAAGCGTTGTCCGGAATTATTGGGCGTAAAGGGTACGTAGGCGGTAATTTAAGTCTGAATTTAAAGGCTGTGGCTCAACCATAGTAAGGTTCAGATACTGGATTACTTGAGTAGATGAGGGGAAAGTGGAATTCCATGTGTAGCGGTGAAATGCGTAGATATATGGAGGAACACCTGTGGCGAAGGCGACTTTCTGGAATCTAACTGACGCTGAGGTACGAAGGCGTGGGGAGCAAACAGGATTAGATACCCTGGTAGTCCACGCAGTAAACGATGAGTGCTAGTTGTCGGGAGTCAAATCTCGGTGACGCAGCTAACGCATTAAGCACTCCGCCTGGGGAGTACGTACGCAAGTATGAAACTCAAAGGAATTGACGGGGACCCGCACAAGCAGCGGAGCATGTGGTTTAATTTGAAGCAACGCGAAGAACCTTTTCACGGCTTGACATATACAGGGATATACTAGAGATAGTATAGTTTCTTCGGAAACTTGTATACAGGTGGCGCATGGTTGTTGTCAGCTCGTGTCGTGAGATGTTGGGTTAAGTCCCGTAACGAGCGCAACCCTTATCTTTAGTTCCCCGCATTTTGGATGGGGACTCTAGAGAGACTGCCGGTGATAAACCGGAGGAAGGTGGGGATGACGTCAAATCATCATGCCCTATATGTCTTGGGCTACACACGTGCTACAATGGTCTGAACAAAGCGCAGCTACCTCGTGAGAGCAAGCGAATCGCATAAAACAGATCTCAGTTCGGATTGTAGGCTGCAACTCGCCTACATGAAGTCGGAGTTGCTAGTAATCGTGGATCAGAACGCCACGGTGAATGCGTTCCCGGGTCTTGTACACACCGCCCGTCACACCATGGGAGTTGGCAATACCCGAA

>gi|284049424|dbj|AB542084.1| Helcococcus ovis gene for 16S ribosomal RNA, partial sequence, strain: H30-Yamagata-071226

TGCTTAACACATGCAAGTTGAACGAGAATTTTTTAATTAATTTCTTCGGAAAGAGATTAAAGAAGGAAAG

TAGCGAACGGGTGAGTAACACGTGAGAAACCTGCCTTTCACAAAGGGATAGCCTCGGGAAACCGGGATTAATACCTTATGACACTTAGATATCGCATGATAATTAAGTTAAAGAATTTCGGTGAAAGATGGTCTCGCGTCTGATTAGCTAGTTGGTAAGGTAACGGCTTACCAAGGCGACGATCAGTAGCCGGATTGAGAGGTTGAACGGCCACACTGGAACTGAGACACGGTCCAGACTCCTACGGGAGGCAGCAGTGGGGAATTTTGCACAATGGGGGGAACCCTGATGCAGCGACGCCGCGTGAACGATGAAGGTCTTCGGATTGTAAAGTTCTGTCCTTAGTGAAGATAATGACTGTAACTAAGAAGCAAGCCCTGGCTAAATACGTGCCAGCAGCCGCGGTAATACGTATGGGGCAAGCGTTGTCCGGAATTATTGGGCGTAAAGGGTACGTAGGCGGTAATTTAAGTCTGAATTTAAAGGCTGTGGCTCAACCATAGTAAGGTTCAGATACTGGATTACTTGAGTAGATGAGGGGAAAGTGGAATTCCATGTGTAGCGGTGAAATGCGTAGATATATGGAGGAACACCTGTGGCGAAGGCGACTTTCTGGAATCTAACTGACGCTGAGGTACGAAGGCGTGGGGAGCAAACAGGATTAGATACCCTGGTAGTCCACGCAGTAAACGATGAGTGCTAGTTGTCGGGAGTCAAATCTCGGTGACGCAGCTAACGCATTAAGCACTCCGCCTGGGGAGTACGTACGCAAGTATGAAACTCAAAGGAATTGACGGGGACCCGCACAAGCAGCGGAGCATGTGGTTTAATTCGAAGCAACGCGAAGAACCTTACCAAGGCTTGACATATACAGGGATATACTAGAGATAGTATAGTTTCTTCGGAAACTTGTATACAGGTGGTGCATGGTTGTCGTCAGCTCGTGTCGTGAGATGTTGGGTTAAGTCCCGTAACGAGCGCAACCCTTATCTTTAGTTACCAGCATTTCGGATGGGGACTCTAGAGAGACTGCCGGTGATAAACCGGAGGAAGGTGGGGATGACGTCAAATCATCATGCCCTATATGTCTTGGGCTACACACGTGCTACAATGGTCTGAACAAAGCGCAGCTACCTCGTGAGAGCAAGCGAATCGCATAAAACAGATCTCAGTTCGGATTGTAGGCTGCAACTCGCCTACATGAAGTCGGAGTTGCTAGTAATCGTGGATCAGAACGCCACGGTGAATGCGTTCCCGGGTCTTGTACACACCGCCCGTCACACCATGGGAGTTGGCAATACCCGAAGCCGTCGAGCTAACCGTTAGGA

>gi|2815221|emb|Y16279.1| Helcococcus sp. 16S rRNA gene, strain CCUG 37441

TGGCTCAGGACGAACGCTGGCGGCGTGCTTAACACATGCAAGTTGAACGAGAATTTTTTAATTAATTTCT

TCGGGAAGAGATTAAAGAAGGAAAGTAGCGAACGGGTGAGTAACACGTGAGAAACCTGCCTTTCACAAAGGGATAGCCTCGGGAAACCGGGATTAATACCTTATGACACTTAGATATCGCATGATAATTAAGTTAAAGAATTTCGGTGAAAGATGGTCTCGCGTCTGATTAGCTAGTTGGTAAGGTAACGGCTTACCAAGGCGACGATCAGTAGCCGGATTGAGAGGTTGAACGGCCACACTGGAACTGAGACACGGTCCAGACTCCTACGGGAGGCAGCAGTGGGGAATTTTGCACAATGGGGGGAACCCTGATGCAGCGACGCCGCGTGAACGATGAAGGTCTTCGGATTGTAAAGTTCTGTCCTTAGTGAAGATAATGACTGTAACTAAGAAGCAAGCCCTGGCTAAATACGTGCCAGCAGCCGCGGTAATACGTATGGGGCAAGCGTTGTCCGGAATTATTGGGCGTAAAGGGTACGTAGGCGGTAATTTAAGTCTGAATTTAAAGGCTGTGGCTCAACCATAGTAAGGTTCAGATACTGGATTACTTGAGTAGATGAGGGGAAAGTGGAATTCCATGTGTAGCGGTGAAATGCGTAGATATATGGAGGAACACCTGTGGCGAAGGCGACTTTCTGGAATCTAACTGACGCTGAGGTACGAAGGCGTGGGGAGCAAACAGGATTAGATACCCTGGTAGTCCACGCAGTAAACGATGAGTGCTAGTTGTCGGGAGTCAAATCTCGGTGACGCAGCTAACGCATTAAGCACTCCGCCTGGGGAGTACGTACGCAAGTATGAAACTCAAAGGAATTGACGGGGACCCGCACAAGCAGCGGAGCATGTGGTTTAATTCGAAGCAACGCGAAGAACCTTACCAAGGCTTGACATATACAGGGATATACTAGAGATAGTATAGTTTCTTCGGAAACTTGTATACAGGTGGTGCATGGTTGTCGTCAGCTCGTGTCGTGAGATGTTGGGTTAAGTCCCGTAACGAGCGCAACCCTTATCTTTAGTTACCAGCATTTCGGATGGGGACTCTAGAGAGACTGCCGGTGATAAACCGGAGGAAGGTGGGGATGACGTCAAATCATCATGCCCTATATGTCTTGGGCTACACACGTGCTACAATGGTCTGAACAAAGCGCAGCTACCTCGTGAGAGCAAGCGAATCGCATAAAACAGATCTCAGTTCGGATTGTAGGCTGCAACTCGCCTACATGAAGTCGGAGTTGCTAGTAATCGTGGATCAGAACGCCACGGTGAATGCGTTCCCGGGTCTTGTACACACCGCCCGTCACACCATGGGAGTTGGCAATACCCGAAGCCGTCGAGCTAACCGTTAGGA
